# Understanding the geometric diversity of inorganic and hybrid frameworks through structural coarse-graining†

**DOI:** 10.1039/d0sc03287e

**Published:** 2020-10-19

**Authors:** Thomas C. Nicholas, Andrew L. Goodwin, Volker L. Deringer

**Affiliations:** Department of Chemistry, Inorganic Chemistry Laboratory, University of Oxford Oxford OX1 3QR UK volker.deringer@chem.ox.ac.uk

## Abstract

Much of our understanding of complex structures is based on simplification: for example, metal–organic frameworks are often discussed in the context of “nodes” and “linkers”, allowing for a qualitative comparison with simpler inorganic structures. Here we show how such an understanding can be obtained in a systematic and quantitative framework, combining atom-density based similarity (kernel) functions and unsupervised machine learning with the long-standing idea of “coarse-graining” atomic structure. We demonstrate how the latter enables a comparison of vastly different chemical systems, and we use it to create a unified, two-dimensional structure map of experimentally known tetrahedral AB_2_ networks – including clathrate hydrates, zeolitic imidazolate frameworks (ZIFs), and diverse inorganic phases. The structural relationships that emerge can then be linked to microscopic properties of interest, which we exemplify for structural heterogeneity and tetrahedral density.

## Abstract 2

A coarse-graining approach enables structural comparisons across vastly different chemical spaces, from inorganic polymorphs to hybrid framework materials.

## Graphical abstract

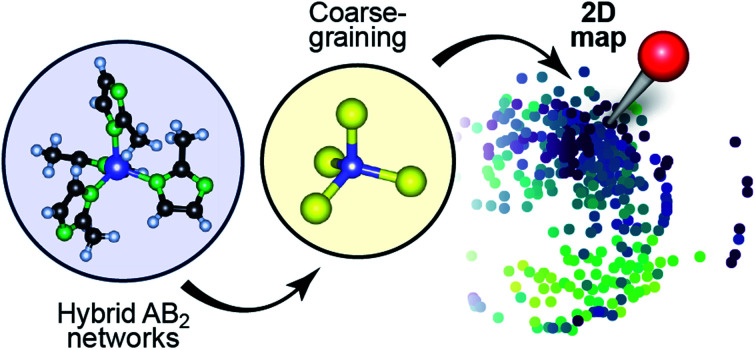

## Introduction

Establishing links between chemical structure and function is a key requirement for developing new materials. The synthetic exploration of solid-state structural space has been documented in extensive databases,^1^ and high-throughput computations and structure prediction are poised to accelerate it even further.^2^ In an aim to navigate this vast space, lower-dimensional representations have been proposed, such as 2D “maps” with chemically informed coordinates, aiming to identify promising synthesis targets.^3^

With machine learning (ML) approaches currently burgeoning in materials chemistry,^4^ it is natural to ask whether they might help with the aforementioned challenges. ML algorithms can handle very large datasets, but are (deliberately) chemically agnostic, and it is not *a priori* clear whether they will discover the same relationships that a trained chemist identifies just by eye. In this context, “unsupervised” ML means that information is sought from a given set of data without labels^5^ – for example, from a mathematical representation of the atomic structure, for which reliable computational tools are now available.^6^

One such representation is given by the Smooth Overlap of Atomic Positions (SOAP) similarity function, or kernel.^6*c*^ This approach builds a neighbour density for any given atom (using “smooth” Gaussian functions) and then evaluates the overlap between pairs of such neighbour densities, making use of an efficient mathematical approach;^6*c*^ a short review is given in the Methods section. SOAP thereby quantifies how similar any two given atomic environments are, on an intuitive scale from zero to one. Initially used for fitting machine-learned force fields,^7^ it was suggested in 2016 that SOAP can be utilised also for visualising chemical space.^8^ Applications to date include known and hypothetical ice structures,^9^ the TiO_2_ polymorphs,^10^ molecular crystals,^11^ and hypothetical zeolites;^12^ an overview including several illustrative examples was given very recently.^13^ Once a SOAP-based structure map has been created, it can be used, *e.g.*, to select the most representative structural motifs in a complex system for computational spectroscopy.^14^

Very recently, zeolites were studied with SOAP-based maps and assessed regarding synthesisability.^15^ These materials are widely described in terms of their topology. Whilst extremely powerful, such approaches do not (by construction) include geometric arguments: two zeolites may differ in their bond lengths and angles yet share identical topologies, or conversely, they may have similar geometric features but different connectivity. SOAP combines all the characteristics of the neighbour environment up to a given cut-off: it thereby cannot reproduce the intuitive classification afforded by the well-known space-group or topology symbols, but in turn gives rise to a comprehensive geometric measure that incorporates bond angles, rings, and other subtleties.^15^

Here, we generalise this approach such that it can make direct comparisons across vastly different families of chemical structures, and thereby we develop a framework in which geometric diversity can be quantified, visualised, and better understood. The key enabling step is the realisation that a density-based metric such as SOAP can be applied equally well to coarse-grained and uniformly scaled representations of chemical structures as to the structures themselves: this allows us to compare compounds with inherently different chemistries and bond lengths. With a long-term aim of discovering (and, ultimately, exploiting) structural relationships, we focus this proof-of-concept study on one notoriously diverse and important family of inorganic and hybrid frameworks: namely, the AB_2_-type networks with tetrahedral-like [AB_4_] environments.

## Results and discussion

**Fig. 1:**
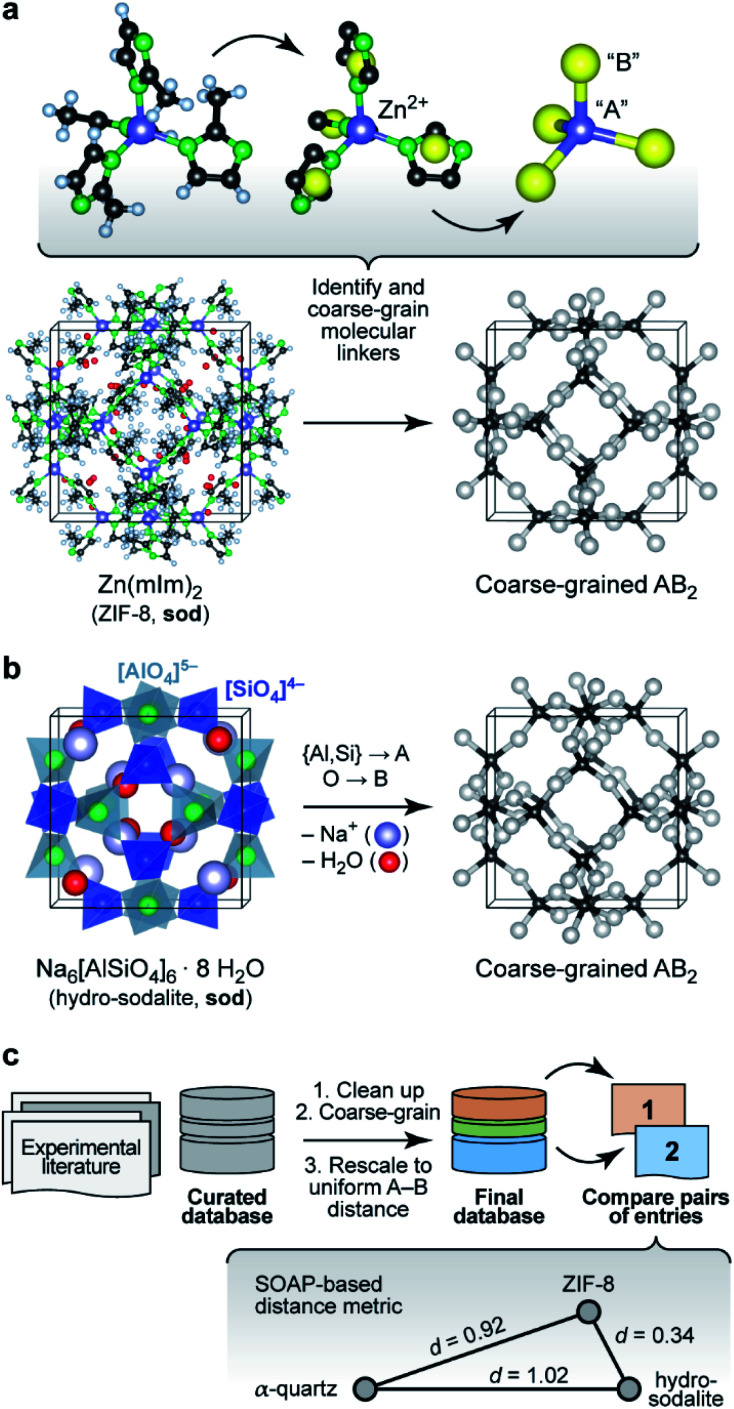
Understanding complex tetrahedral inorganic and hybrid structures by reducing them to the underlying AB_2_ networks (“coarse-graining”). (a) The prototypical zeolitic imidazolate framework, ZIF-8,^17^ can be reduced by placing dummy atoms (represented by yellow spheres) at the midpoint of the N⋯N contact inside a single methylimidazolate (mIm) linker. The resulting simplified (“coarse-grained”) structure contains an “A” atom for each Zn^2+^ position, and a “B” atom for each linker: we obtain an open AB_2_-type structure with four- and six-membered rings (sod vertex symbol, using the notation pioneered by O'Keeffe and others; ref. 28). (b) The crystal structure of the inorganic mineral hydro-sodalite^19^ is based on the same framework topology. To illustrate this relationship, we remove the (partly occupied) Na sites and the water molecules within the framework, and we reduce the Al and Si cation sites to a single “A” atom. This way, we arrive at a representation that looks very similar to that of ZIF-8 above. There are still differences in the orientation of the individual tetrahedra, and characteristically different absolute A–B distances, which need to be re-scaled for proper comparison. (c) Overview of the workflow in the present study, with database building, processing, and then analysis. The inset illustrates the concept of SOAP-based distances, *d*, for a set of three structures: ZIF-8 and hydro-sodalite (shown above) are quite similar in their coarse-grained and re-scaled representations; α-quartz is very different from both. Note that rather than the absolute values, it is the relative distances between the points which are most meaningful (see also Methods section). Structures were visualised using VESTA.^29^

We start by noting that whilst comparisons across AB_2_ structures have been eminently useful,^16^ they have normally been limited to individual aspects either of the structure (say, the A–B–A angles) or topology (thereby removing subtleties of the structure itself). For example, zeolitic imidazolate frameworks (ZIFs), such as ZIF-8 (Fig. 1a),^17^ have been discussed in terms of the analogy to Si–O–Si angles in SiO_2_ polymorphs.^18^ We now use a computer algorithm for the same task: placing “dummy” atoms at the midpoint between those (nitrogen) atoms that connect to the Zn^2+^ centres, as shown in Fig. 1a (note that this is not the same as the centre of mass of the entire linker, which would distort the resulting angles for larger ligands, such as benzimidazolate).

A classical inorganic example of a more complex AB_2_ solid is hydro-sodalite (Fig. 1b).^19^ In this case, we need to remove intra-framework Na^+^ ions and water from consideration; our workflows and code are designed to carry out this “clean-up” step in a largely automated fashion (ESI†). We also discard the chemical distinction between two different cation sites – now represented by a single “A” dummy atom – but retain any geometric differences in their local environments. This idea of increasing the granularity of the structure is in analogy to how coarse-graining approaches are used for molecular-dynamics simulations that traverse atomistic and larger length scales,^20^ and how secondary building units (SBUs) are identified in inorganic solids and metal–organic frameworks.^21^ We refer to the resulting approach, including removal of guests, coarse-graining, and re-scaling, as “cg-SOAP” in the following.

To test this idea on a much wider basis of experimentally validated structures, we assembled a dataset which includes diverse families of AB_2_-like materials, including zeolites, ices, and chain-like inorganic structures such as BeCl_2_. Among the data sources, we point out a review article on ZIFs by Yaghi and co-workers,^18*a*^ a report on cadmium-based imidazolate frameworks (“CdIFs”) by Tian *et al.*,^22^ and a study of polymorphism in Zn(CN)_2_ by Chapman and co-workers.^23^ More structures were collected from the Cambridge Structural Database^1*b*^ and the IZA Database of Zeolite Structures.^1*e*^ Key information about this dataset is collected in Table 1, and full data and references (including justification for any structures that have been discarded, *e.g.*, because they contain non-tetrahedral environments) are given as ESI.†

**Table 1:** Overview of the curated database of AB_2_ structures, and their coarse-grained representations, as developed in the present work. Details are given in the ESI

Material class	A site	B site	Entries
Zeolites/AlPOs	Si, {Al, P}, various others	O	245
Silica	Si	O	9
Cyanides	Zn	(CN)	4
Other inorganics	Be, Zn, Si, {Li, Co}	Cl, Cl, S, (CO), respectively	7
Clathrates	O	H	8
Disordered ices	O	H	10
Ordered ices	O	H	6
ZIFs	Zn, Cd, Hg, Co, Fe, Cu, In^a^	Organic	70
CdIFs	Cd	Organic	12
BIFs	{Li, B}, {Cu, B}	Organic	6
TIFs	Zn	Organic	6

a The indium compound (ref. 26) is an example for a different oxidation state (+3) being accommodated by a more complex organic counterpart. In this specific case, a delicate combination of structure-directing agents was used: the unit cell contains 4,5-imidazoledicarboxylate (Himdc) linkers, protonated amines balancing the charges, and three different solvents.^26^ All this complexity is identified and reduced by our approach, transforming the structure to its fundamental AB_2_ network.

Once the coarse-graining is done, one key step remains before these very different chemistries can be compared using SOAP: we re-scale the structures such that the shortest A–B distance in any given structure is the same (here, 1.0 Å)^24^ – an idea that originated in the field of chemical topology.^25^ This is a step of key importance, because otherwise the overlap of neighbour densities will be necessarily diminished as soon as there are different A–B distances (Fig. S5 in the ESI†). The workflow on which the following analysis is based is shown in Fig. 1c.

The SOAP kernel is a similarity measure between two atomic environments, *k*(*α*,*β*),^6*c*^ on a scale from 0 to 1, obtained here using the openly available DScribe implementation.^27^ Details are given in the Methods section. In short, averaging over all combinations of A-site environments *α* in the *i*-th unit cell in our database and *β* in the *j*-th, we obtain a per-cell similarity, *k̄*(*i*,*j*). With this, one may then define a geometric distance (dissimilarity) between the *i*-th and *j*-th unit cell as1
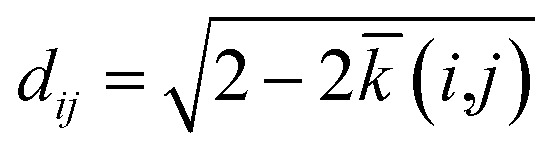
to satisfy the triangle inequality (Fig. 1c).^8^

**Fig. 2:**
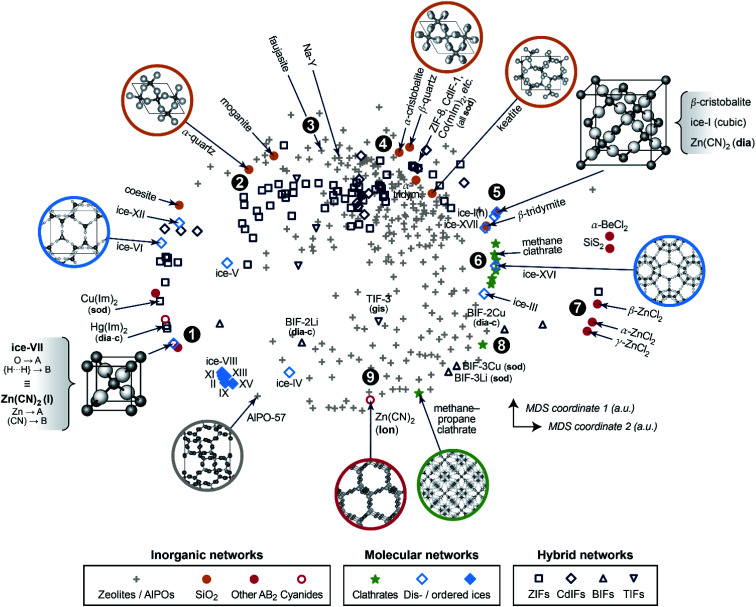
A two-dimensional map for inorganic and hybrid tetrahedral structures. The closer two points are, the more similar the corresponding structures, and *vice versa*. This visualisation is based on a structural dissimilarity (distance) metric, using the SOAP kernel to compare coarse-grained and re-scaled structures (*cf.*Fig. 1c), and on embedding by multi-dimensional scaling (MDS). Different symbols are used for the various types of inorganic, molecular, and hybrid networks that are all part of our database. Points of interest are marked as 1, 2, and so on, and discussed in the text in this order.

We now progress to a much larger structure map that represents distances, obtained from eqn (1), between many different chemical systems and structure types. To visualise these distances, we use a basic unsupervised ML approach, multi-dimensional scaling (MDS) – a projection into a 2D space which directly takes distances as input and can thus be coupled to SOAP in a straightforward way.^10,14^ Our map is shown in Fig. 2 and spans all entries of our manually curated database (*cf.*Table 1), classified according to inorganic (*e.g.*, SiO_2_ polymorphs), molecular (*e.g.*, ice networks), and tetrahedral hybrid networks, *viz.* ZIFs and related cadmium-, boron-, or other cation based tetrahedral imidazolate frameworks (“TIFs”). We follow the naming conventions in the existing literature, accepting that the abbreviations will not always be entirely unambiguous – *e.g.*, for cadmium-based species: Cd(Im)_2_-**dia-c** was labelled as a “ZIF” in ref. 18*a*, whereas Cd(mIm)_2_-**sod** was initially reported as “CdIF-1” shortly thereafter.^22^

In the 2D space of Fig. 2, structures that are similar appear close together, and structures that are dissimilar are further apart. Some material classes are widely distributed throughout the space which is spanned by the map, with the widest absolute distribution found for the zeolites (“+”). Hybrid frameworks (blue symbols) occupy some of this space, but distinctly not all of it; SiO_2_ polymorphs and disordered ices (such as the common ice-I) are widely spread as well, whereas ordered ices are clustered closely together in the bottom left area. In addition to the absolute distribution across the map, we may quantify the relative distribution for each materials class, by which we mean the standard deviation of how far points are from their respective centre of mass – normalised such that the SiO_2_ polymorphs have a relative distribution of 1.0. ZIFs (zeolites) attain values of 1.20 (1.04), respectively. On the other hand, the ordered ices have a relative distribution of only 0.06, consistent with lower geometric flexibility in their strongly directional hydrogen-bonded networks.

We now walk through this map in clockwise direction, having labelled some more specific locations of interest with boldface numbers. In the lower left part, there is a point where two structures coincide exactly in the 2D map (1). One is disordered ice-VII, where we reduce the O–H⋯H–O bridge (with both hydrogen sites half-occupied) to an A–B–A link. The other is the ambient polymorph of zinc cyanide, for which we also reduce the Zn⋯C≡N⋯Zn motif to a symmetric A–B–A link because of head-to-tail orientational disorder of the CN^−^ linkers. Both phases are based on the same anticuprite structure, with no internal degrees of freedom; hence the two corresponding points coincide perfectly. LiCo(CO)_4_ adopts a lower-symmetry variant of the same structure type,^30^ with the CO ligand closer to Co than Li – its midpoint is shifted along (*x*, *x*, *x*) from *x* = 0.25 to 0.241. That structure is therefore almost, but not exactly, in the same location on the cg-SOAP map in Fig. 2.

Moving up past other disordered ices, the silica polymorphs begin to appear in the upper left part of the map in Fig. 2. We illustrate α-quartz, the stable form at ambient conditions (2). Not many hybrid frameworks (blue) are found in its immediate vicinity, from which we infer that its particular geometry is relatively unusual in the wider context of AB_2_ networks. We move clockwise past more open framework structures, *viz.* the faujasite and Na–Y cages (3), and we find β-quartz near the top of the map (4). In the immediate vicinity, there is then a rather largely populated cluster of ZIFs and related structures (all represented by dark blue symbols). Of note is the cadmium-based framework, CdIF-1, which has **sod** topology (*cf.*Fig. 1b), and is therefore located alongside other sodalite-type ZIFs.

β-Cristobalite is another high-symmetry structure with no internal degrees of freedom, located in the upper right part of the map (5). After coarse-graining and re-scaling, cubic ice-I and **dia**-Zn(CN)_2_ occupy exactly the same location; hexagonal ice-I is very close. We find a region of clathrate hydrates (6), related to the “empty” frameworks of the low-density ices III and XVI, reflected in close proximity in the cg-SOAP map. Separated clearly from the main area of the map, there is then an “island” of inorganic structures on the right-hand side (7): *e.g.*, SiS_2_, which features chains of edge-sharing tetrahedra, very different from the compositionally homologous SiO_2_ polymorphs in which all tetrahedra are corner-sharing.

In the lower right part of Fig. 2, we find again more open frameworks. Of note are the boron-based BIFs (8), which contain Li^+^ or Cu^+^ cations in combination with B^3+^, and therefore are aliovalent equivalents to ZIFs (M^2+^).^31^ We re-iterate that even though we reduce the cation sites to a single type of “A” dummy atom, we do retain the relative differences in bond lengths around M^+^*vs.* B^3+^; therefore, the BIF-3 frameworks are not near other **sod** structures. Finally, near the bottom of the cg-SOAP map in Fig. 2, we point out another form of zinc cyanide (9), emphasising the large variety of polymorphs that is accessible to a single system.^23^ This particular one adopts the same topology as hexagonal ice-I (**lon**) – but in the Zn(CN)_2_ structure, the metal⋯cyanide distances are very dissimilar, about 1.6 and 2.0 Å respectively, and the data point is therefore away from ice-Ih in the 2D map of Fig. 2. In the context of cyanides, we mention the even larger structural diversity in Prussian blue analogues:^32^ this exemplifies a limit of our method in that it needs discrete positions for the “B” grains, and it cannot capture longer-range correlated disorder beyond the pairwise SOAP cut-off distance. Another limitation of the present approach is given by large and highly directional linkers such as [Au(CN)_2_]^−^ which lead to incorrect A⋯A contacts, shorter than the shortest A–B ones, when dense interpenetrating networks are considered. An example, with six independent interpenetrating nets, is the structure of Zn[Au(CN)_2_]_2_;^33^ related issues will often occur for MOFs, where interpenetration is commonplace.

**Fig. 3:**
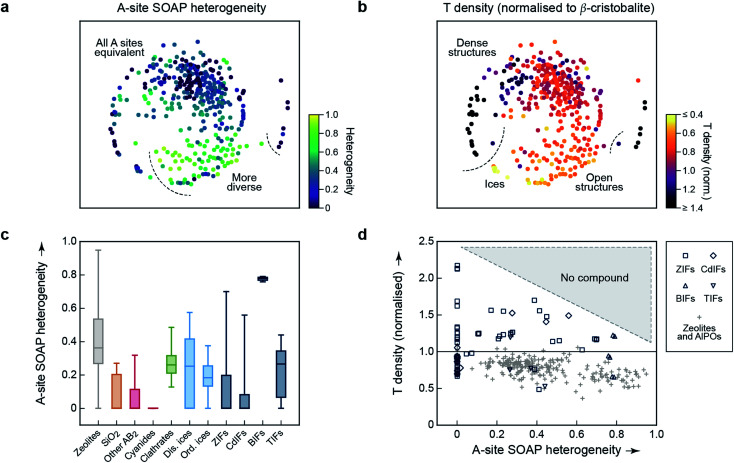
Geometric diversity in tetrahedral networks analysed with our methodology. (a) A-site SOAP heterogeneity (that is, a measure for how dissimilar cationic environments are within a given structure), colour-coded on the 2D map from Fig. 2. (b) Tetrahedral (“*T*”) density, given relative to β-cristobalite, colour-coded on the same map. (c) A more quantitative analysis of the A-site SOAP heterogeneity, in which the data have now been collected according to the different categories. The box plots indicate the distribution of data: the boxes range from the 25th to the 75th percentile (with a horizontal line indicating the median), and the whiskers indicate the full range of data points. For boxes without a visible horizontal line, the median is zero. (d) Connecting both quantities for framework materials and zeolites: the *T* density for each corresponding entry of our database has been plotted as a function of A-site heterogeneity. There is a class of low-density zeolites (“+”) that correlate with large A-site heterogeneity (>0.6), but dense structures require local homogeneity.

An important aspect of a materials map is that it should be able to be correlated with relevant properties.^3^ The first quantity for which we test this question is again concerned with structural diversity. In Fig. 1c and 2, we had used an averaged metric to compare different unit cells with one another – but SOAP can also be used to compare individual atoms within one and the same structure. We may therefore use it to assess the question of how diverse the different A-sites in any given structure are, which we call “A-site SOAP heterogeneity”: a value of zero means that all A-site environments (normally, metals) are geometrically equivalent, and a higher value indicates a higher degree of diversity – *e.g.*, in the BIFs, where different aliovalent cationic species occupy the A site, as mentioned above. This information can be visualised in a colour-coded version of our map, which is shown in Fig. 3a.

SOAP maps are beginning to be used to identify properties of application interest.^15^ In the context of the present work, a central such property is the tetrahedral (“*T*”) density: this is the simplest proxy for possible usefulness in catalysis, because low *T* densities indicate the presence of voids in the framework, which could be used for the absorption, diffusion, and transformation of guest molecules – noting that the *T* density of the re-scaled framework need not directly correspond to the accessible pore volume, nor indeed to the density of catalytically active sites. We show a colour-coded version of our map, illustrating the *T* density, in Fig. 3b. Again, there are clearly different regions, evidencing the physical significance of the initially chemically agnostic unsupervised ML approach. The two colour-coded maps also show an inherent characteristic of the 2D embedding: it needs to balance all structural aspects, and therefore the very dense networks at the bottom left are close to the very open, ordered ices (Fig. 3b). We presume that this is linked to the A-site heterogeneity, which is low in both groups, and prohibits the ices from being in the lower right region with its more diverse A sites (Fig. 3a). It is also an indication of the need for any embedding scheme to balance local structure (bringing similar points close together) with aspects of the global structure (keeping dissimilar points far apart in the 2D map).

The embedding of high-dimensional distances in 2D invariably leads to the loss of some information. It is therefore useful, in addition to the map, to look quantitatively at similarities and properties independent from where a given material is located in the 2D map. We quantify the distribution of A-site SOAP heterogeneities, separately for the different materials classes, in Fig. 3c. Some of the SiO_2_ polymorphs include locally heterogeneous environments (the monoclinic structures of moganite, with heterogeneity 0.27, and coesite, 0.21, are of note) – but most of them do not, and neither do most other inorganic AB_2_ structures. In clathrates, on the other hand, we do not find any fully homogeneous structure (even the minimum value being >0). Disordered ices are overall more heterogeneous than ordered ones. Among the framework materials, CdIFs are the least locally heterogeneous, which is perhaps surprising given the large ionic radius and polarisability of Cd^2+^; BIFs show a large, and narrowly distributed, heterogeneity in Fig. 3c, as expected due to the presence of two different cationic species.

Finally, the information content of Fig. 3a and b can be combined to study correlations between different property indicators. We do this for the subset of hybrid frameworks and zeolites (Fig. 3d). There is a number of fully locally homogeneous structures, mainly composed of the different hybrid framework materials (at a heterogeneity value of *x* = 0), but there are also two distinct regions of heterogeneity (up to *x* = 0.6 and beyond it, respectively), dominated by zeolite structures (“+”). Generally, Fig. 3d reveals that all heterogeneous tetrahedral networks studied have low density, and conversely all dense networks are homogeneous; there is a distinct region where no compounds have been experimentally observed, indicated by shading. It appears reasonable to assume that a too large geometric mismatch will tend to keep dense structures from forming. When aiming to design new low-density materials, one might therefore attempt to introduce and tune A-site heterogeneity. The latter can be achieved experimentally, *e.g.*, by exploiting solid-solution chemistry, both regarding isovalent or aliovalent cations, and combinations of different linkers.

## Conclusions

We have shown how structural relationships across diverse material families can be understood by combining the idea of coarse-graining and scaling atomistic structure with a suitable atom-density based similarity metric (here, SOAP). Our study has built on experimentally characterised structures and a carefully curated database of those, but similar approaches may now be extended to even larger sets of data: to hypothetical zeolites,^34^ hybrid perovskites,^35^ or to a more extensive range of MOFs,^36^ for example. Our approach is chemically agnostic on purpose (allowing us to compare, say, ices with zeolites) – although we note that the purely geometric SOAP kernel can be amended with terms that depend on the atomic numbers, or even with entirely different kernel definitions that capture, *e.g.*, similarities in the electronic structure.^37^ Such combined models could then extend to application-related properties which are determined by geometry and chemistry (*e.g.*, catalytic activity). In regard to visualisation tools, we used one of the simplest (*viz.*, MDS), which already leads to appreciable results, but one might couple our approach to other, more involved dimensionality-reduction schemes such as the popular sketch-map scheme^38^ or *t*-stochastic neighbour embedding^39^ which are also beginning to be used with SOAP,^8,15^ and to openly available implementations which are beginning to emerge.^13,40^ To this end, our database of all coarse-grained representations will be made openly available online upon publication of this work, with the hope to enable future work in the community.

## Methods

SOAP measures the overlap (that is, the similarity) of pairs of atomic environments,^6*c*^ here denoted *α* and *β*. To describe the environment of an atom *α*, an atomic density, *ρ*_*α*_(**r**), is constructed by placing Gaussian functions, of broadness *σ*, on the atomic positions. The neighbour density is then expanded in a local basis set of suitable radial functions, *R*_*n*_, and spherical harmonics, *Y*_*lm*_:2
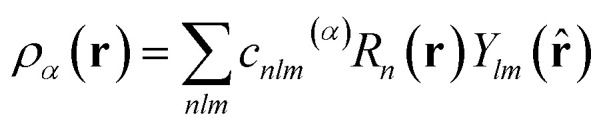
up to a given *n*_max_ and *l*_max_. This way, by collecting the combination coefficients, *c*_*nlm*_^(*α*)^, into a power spectrum vector, **p**_*α*_, one may then evaluate the similarity of two environments, *α* and *β*, by means of a simple dot product:

3 *k*(*α*,*β*) = [**p**_*α*_·**p**_*β*_]^*ζ*^,

where exponentiation by *ζ* controls the sharpness of the distinction between the two environments.^6*c*^

We computed SOAP vectors using the polynomial basis functions implemented in DScribe (https://github.com/SINGROUP/dscribe/),^27^ an expansion of the atomic neighbour density (eqn (2)) up to the available maximum of *n*_max_ = 10, *l*_max_ = 9, and a radial cut-off of 2.5 Å and a smoothness of *σ* = 0.2 Å (note that both values refer to re-scaled structures and thus include next-nearest-neighbour environments). We used a relatively large exponent for the “sharpness” of the kernel (eqn (3)), *viz. ζ* = 8, compared to a typical choice of *ζ* = 4 for ML potential fitting.^7*a*^ We note that the SOAP implementation in DScribe differs slightly from that in the original GAP code (available at http://www.libatoms.org/gap/gap_download.html), *e.g.*, using fewer descriptor vector entries in multispecies systems, but these differences are not expected to affect our conceptual findings or the interpretation of cg-SOAP maps. For the same reason, no numerical coordinates are given in the map in Fig. 2, similar to previous work.^8,10,24^

We obtained the per-cell similarity, *k̄*(*i*,*j*), as4
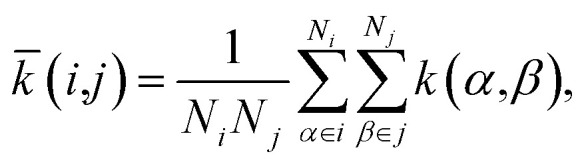
where *α* (*β*) runs over all A sites in the *i*-th (*j*-th) cell, respectively, and the coarse-grained B sites are included in the respective neighbour densities of the A-sites *α* and *β* (details of the A- and B-site species are given in Table 1). The handling of structures was aided by the freely available Atomic Simulation Environment (ASE).^41^ We note that different ways of defining averaged kernels (*e.g.*, by averaging over the SOAP expansion coefficients rather than averaging over the kernel values themselves) have been proposed;^8,10*a*^ the optimised choice of these definitions for cg-SOAP maps will be the subject of future, more technical work.

MDS maps were generated using the freely available scikit-learn package.^42^ The technique performs a least-squares minimisation of the stress, defined as5
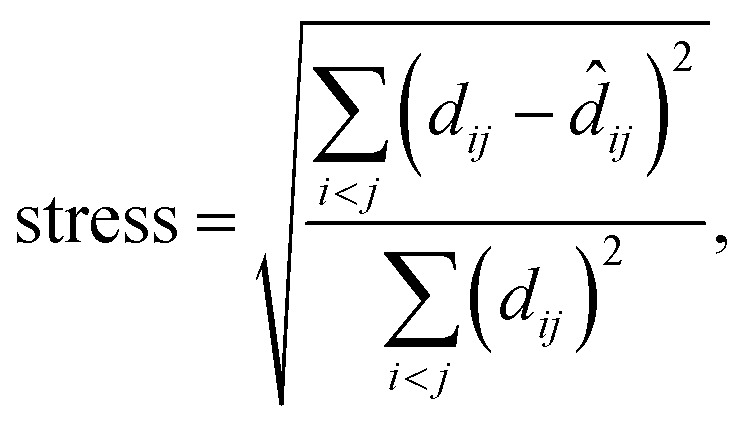
where *d*_*ij*_ is the SOAP distance between the *i*-th and *j*-th atomic environment in high-dimensional space (eqn (1)), and *d̂*_*ij*_ is the distance of the corresponding points in the embedded (here, 2D) representation. The stress is zero if the original distances are fully respected. We obtained a stress value of 0.251, with convergence defined by a maximum change of 10^−5^. There is, hence, an appreciable loss of some part of the high-dimensional information, but this does not impair the validity of our 2D map (evidenced, *e.g.*, by the visible correlations in Fig. 3a and b). Taking the SOAP-based distance as input directly, MDS does not require specific engineering of features or definition of other hyperparameters. It does require a choice of random seed for the minimisation, but we confirmed that different choices of this seed did not change the appearance of the map outside of numerical differences.

## Data availability

A list of all structures (including database accession codes) that form the basis for this work is given as ESI.† Further data supporting this work are available at https://doi.org/10.5281/zenodo.4118220.

## Conflicts of interest

There are no conflicts to declare.

## Supplementary Material

SC-011-D0SC03287E-s001
