# Supplementary material for: Understanding the geometric diversity of inorganic and hybrid frameworks through structural coarse-graining
Source: Chem Sci. 2020 Oct 19;11(46):12580–7. doi: 10.1039/d0sc03287e (PMC8162807; doi:10.1039/d0sc03287e)
Supplement: SC-011-D0SC03287E-s001 [file SC-011-D0SC03287E-s001.pdf]

Electronic Supplementary Material (ESI) for Chemical Science.
This journal is © The Royal Society of Chemistry 2020

# Electronic Supplementary Information (ESI) for

## Understanding the geometric diversity of inorganic and hybrid frameworks through structural coarse-graining

Thomas C. Nicholas,[a] Andrew L. Goodwin,[a] and Volker L. Deringer*

[a]Department of Chemistry, Inorganic Chemistry Laboratory, University of Oxford, Oxford OX1 3QR, UK

*volker.deringer@chem.ox.ac.uk

This ESI document contains additional information about the procedures used for:

- **Selecting** structural data (p. S2)
- **Processing** and "cleaning up" structural data (p. S3)
- **Coarse-graining** structural data for different material systems (p. S4)
- **Re-scaling** structural data as input for cg-SOAP, and why this is needed (p. S7).

In addition, this document contains additional information and references for all entries of the database; structures which are included ("y") and structures *not* included for specified reasons ("n") are listed in tables as follows:


- **Table 1** (p. S8): ZIFs and related hybrid framework materials
- **Table 2** (p. S11): Silica ($SiO_2$) polymorphs
- **Table 3** (p. S12): Aluminophosphates (AlPOs)
- **Table 4** (p. S18): Ice structures
- **Table 5** (p. S19): $Zn(CN)_2$ polymorphs
- **Table 6** (p. S20): Clathrate structures
- **Table 7** (p. S21): Other inorganic structures
- References for all these are given at the end of the document, starting on p. S22.

## Selection of structural data

Creating the database for this work consisted of two stages: compiling comprehensive lists of experimental structures representing chemical families typified by 4-fold connected networks, and curating the resulting database according to strict criteria. These stages are described in the following subsections. References for all original data are given starting on p. S8.

**Data sources.** A 2009 review of all synthesised ZIFs provided a list of hybrid inorganic-organic materials for inclusion (DOI: 10.1021/ar900116g). In the same year, a family of cadmium imidazolate frameworks ("CdIFs") were also reported by Tian *et al.* (DOI: 10.1002/chem.200902729) and these structures are also included. All zeolites, AlPOs, and related structures in the Database of Zeolite Structures are included (http://www.iza-structure.org/databases/). The 9 distinct, naturally occurring crystalline silica polymorphs and the 17 experimentally realised ice polymorphs are also included. The cyanides were taken from a comprehensive report of various porous polymorphs of $Zn(CN)_2$ (DOI: 10.1021/ja4012707). The remaining inorganic and clathrate structures were chosen manually and taken from either the Cambridge Crystallographic Data Centre (CCDC) or the Inorganic Crystal Structure Database (ICSD).

**Selection criteria.** Each entry was manually checked, and only accepted into the curated database if:

(i) the framework has $AB_2$ stoichiometry;

(ii) each A site environment is tetrahedral;

(iii) the network is fully connected; and

(iv) the A–B–A bonding is well represented when the B site species is modelled as a single point.

It should be noted that this final point (iv) is a limitation of the present method. For example, the highly directional bonding associated with ligands in $M[Au(CN)_2]_2$ ($M$ = Zn, Co) or large MOF ligands, in which the coordinating ligands atoms are greatly separated in space, are poorly represented by a single point. As such, these structure classes were not considered for the present work.

## Cleaning up structures

Cleaning up the structures involves the removal of guest species (solvent molecules, (organic) SDAs, and counter-ions) and averaging partial-occupation and thermally disordered sites.

**Guest atoms.** Following the identification of the A and B site atoms of structures, all other atomic species are removed. This approach works provided that, for materials in which O is a constituent member of the framework, the pores do not contain O atoms.

Two scenarios are commonly found in which this is not the case. Firstly, if the guest species contains any O atoms (for example, if a solvent such as methanol resides in the pores). Secondly, in the crystallography of porous frameworks the electron density associated with guest species within the pores is often delocalised, and thus not resolved. This is often modelled as clusters of (artificial) O atoms.

Zeolite gismondine demonstrates both of these situations, and the guest removal procedure is illustrated in Figure 2.2. Guest O species are removed by two sequential "tidying" algorithms. The first removes clusters of O atoms by iterating over all O atom indices, and if a second O atom is found within a distance of 1.8 Å, both are removed (**Fig. S1**; step **1**). Any remaining guest O atoms are then removed by iterating over all remaining O atom indices, and if no A site nodes lie within a sphere of radius 1.8 Å, about the O atom, it is removed (Fig. S1; step **2**). The distance, 1.8 Å, was chosen based on typical Si–O and Al–O bond lengths (between 1.58 and 1.78 Å), and by manual inspection of the structures concerned. (The specific cut offs used may have been changed manually from these values for a given structure.)

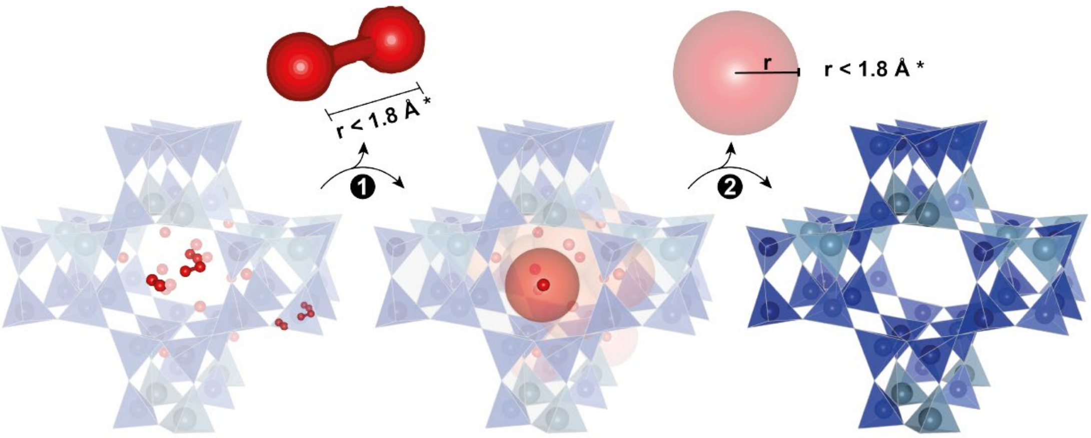


**Fig. S1** Removal of interstitial oxygen atoms, exemplified for the zeolite gismondine.

**Disordered sites.** In many crystal structures atoms have partial occupancy, either as a result of thermal motion or occupational disorder; in contrast, the approach presented here requires well-defined A and B sites with unity occupation. To deal with disordered sites, we iterate over all indices of the framework atom species concerned and, if a second atom of the same species is found within a distance $r$ (where $r = 0.9$ Å for N atoms, else $r = 0.6$ Å), a new atom of the same atomic species is placed at the average position. The original two atoms are then removed.

These two cut-off distances were chosen to strike a balance between not missing any disordered sites while also avoiding mistakenly averaging closely situated atoms. A separate distance was used when considering N disorder because it was usually a result of rotational disorder in the imidazolate ligands, and therefore the separation of the two sites was larger, relative to thermal vibrational disorder.

## Coarse-graining

The coarse-graining procedures identify and equalise all A sites to the same atomic species, and identify all B sites and place a dummy atom at the centre of the bonding interactions. All other atoms are then removed. Specific coarse-graining routines have been developed that identify and reduce the different types of frameworks to the fundamental $AB_2$ units.

**Inorganic networks.** The cyanides (as well as $LiCo(CO)_4$) are coarse-grained by identifying the midpoint of each CN (or CO) ligand, and placing the dummy B site atom there. Then all A site atoms (Li, Co, and Zn) are equalised to the dummy A atom. The coarse-graining procedure for the remaining inorganic networks is illustrated for gismondine in **Fig. S2**. First, the framework atoms are identified. We assumed that any Si, Al, P, and O atoms are a part of the underlying framework, and included an option to enter the atomic symbol manually for any node not included in this list. Following the identification of the underlying inorganic 4-c network atoms, all other atoms are removed. Any O atoms found within the pores are then removed by the tidying algorithms. Finally, all O atoms (B sites) are labelled with the dummy atom.

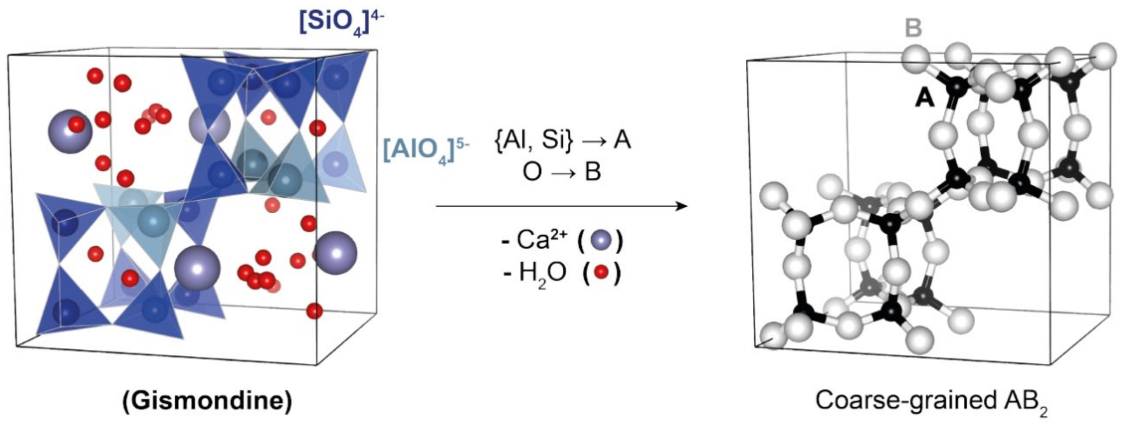


**Fig. S2** Clean-up and coarse-graining of inorganic structures, exemplified for the zeolite gismondine: details of the clean-up step have been illustrated in Fig. S1, but we here show additionally the reduction to the fundamental $AB_2$ network by equalising the {Al, Si} A-sites.

**Ices and clathrate hydrates.** The ices incorporated in the curated database are classified as either H-ordered or H-disordered structures. The H-ordered ices have unity occupation for all protons and therefore have the correct $AB_2$ stoichiometry. All protons are replaced by the B site dummy atom. The H-disordered ice and clathrate hydrate structures all have half-occupancy proton positions which are dealt with separately.

As illustrated by ice IV in **Fig. S3**, for a generic algorithm to be implemented it cannot be assumed that each pair of closest protons is necessarily from different O nodes. To overcome this, the constituent atoms of each tetrahedron are identified by taking the nearest four H neighbours to each O node. Then, iterating over all pairs of tetrahedra, if the distance between any two protons from different tetrahedra is less than 1.6 Å, a B-site dummy atom is placed at the mid-point and the protons are removed.

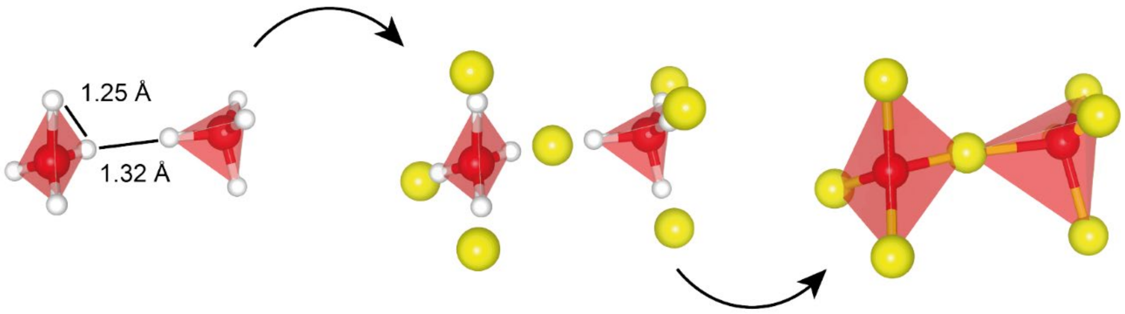


**Fig. S3** The coarse-graining approach for the H-disordered ices and the clathrate hydrates, in which four H atoms surround a central O, each with an occupation of ½. First, these tetrahedra are identified (red). The "dummy" B atom (yellow) is then placed at the mid-point between nearest-neighbour protons from *different* tetrahedra. Finally, all protons are removed.

**Hybrid networks.** The hybrid networks in the database all have imidazolate-derivative organic linkers. The main framework atoms are defined as the metal nodes (A sites) and the N–C–N motifs in each imidazole linker (B sites).

To identify the B sites automatically, our algorithm first identifies the N–C–N motif (**Fig. S4**a). This is achieved by iterating through the C atoms and identifying if there are N atoms within a sphere of radius, 1.5 Å, centred on the C atom. If three N atoms are found it is assumed the imidazolate-derivative has a $NO_2$ side-chain. The nitrate N atom is then distinguished from the bonding N atoms by searching for the two O atoms anticipated within a sphere of radius, 1.4 Å. Once two bonding N atoms have been identified, the B site dummy atom is placed at the midpoint of the N···N contact. The structure is then coarse-grained by removing all but the A and B site dummy atoms, as illustrated for ZIF-74 in Fig. S4b–c.

There are two reasons for taking the midpoint of the N···N contact as the B site position instead of the centre of mass of the ligand. Firstly, large side-chains on the imidazolate ligand would skew the centre of mass away from the intuitively assumed midpoint of the link. (This effect would be exacerbated by thermal disorder in the ligand because the present approach does not assign fractional weights). This would result in a distortion to the bond angles and lengths, giving an unfaithful representation of local structure. Secondly, it ensures a uniform placement of the B site dummy atom in chemically modified series with different side-chains.

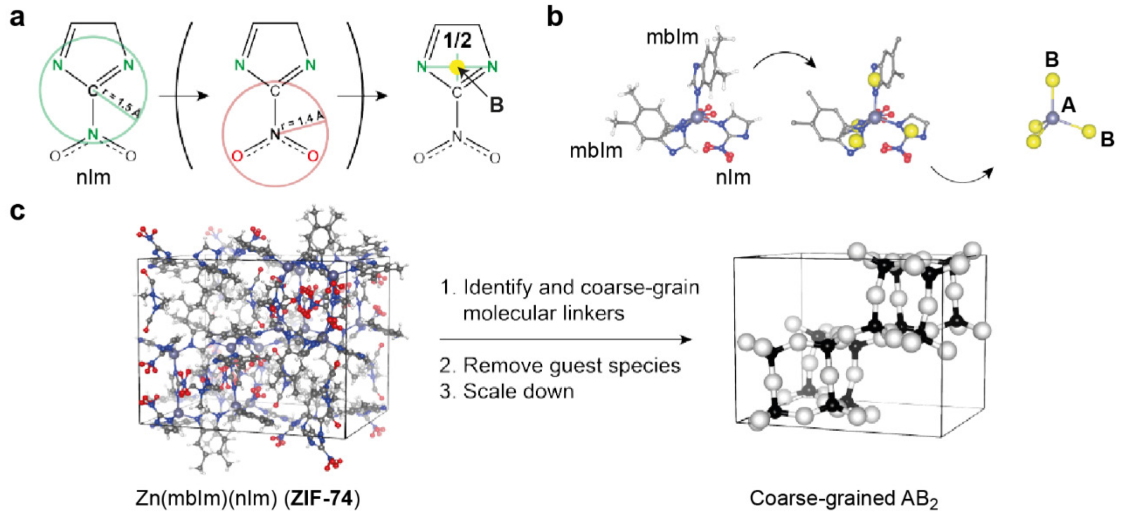


**Fig. S4** The coarse-graining approach for hybrid structures. Panel (**a**) demonstrates a general scheme for identifying the (nitro-)Im ligands, as discussed above. Panels (**b**) and (**c**) illustrate the procedure for Zn(mbIm)(nIm) (ZIF-74) in which two different linkers are coarse-grained.

## Rescaling

Finally, all structures are scaled down to a uniform minimum bond length ($r_0$). This scaling is of key importance, because it enables the comparison of structures with inherently different chemistries and bond lengths. The scaling is achieved by calculating the distance between all atoms, and using ASE's `set_cell` function – which allows one to scale the dimensions of the unit cell and the atomic positions proportionately – to scale the structures by dividing by the minimum bond distance found (such that in all re-scaled structures, the new minimum distance is $r_0$ = 1 Å). **Fig. S5** illustrates the need for doing this by comparing (cg-) SOAP distances between pairs of relevant structures: geometric relationships are only properly recovered if the coarse-graining *and* re-scaling steps are applied.

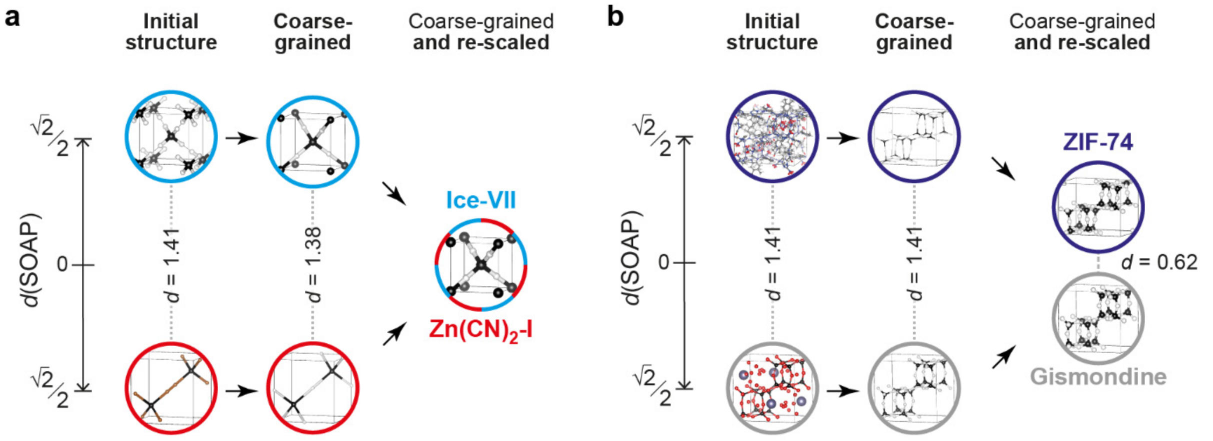


**Fig. S5** Examples illustrating the requirement for both the coarse-graining and the re-scaling of structures in our approach. (**a**) Ice-VII and the $Zn(CN)_2$-I polymorph both crystallise in an anticuprite-like structure. The initial structures are fully dissimilar when using a chemically selective SOAP kernel (i.e., one that distinguishes between, say, O and Zn atoms on the A-site). The coarse-grained ($AB_2$) representations are still highly dissimilar ($d$ = 1.38), because the A–B distances are very different (note that for the analysis of structures before re-scaling, the kernel cut-off and smoothness have been adjusted accordingly to $r_{cut}$ = 6.0 Å and $\sigma$ = 0.4 Å, respectively). Subsequent re-scaling to a uniform A–B distance, however, shows that the resultant structures are identical (with a cg-SOAP distance of zero). (**b**) Same for the isoreticular structures of ZIF-74 and gismondine, which have served as examples in the figures above.

The Zeolitic Imidazolate Frameworks review paper by Phan et al.[1] provided a foundation dataset for the ZIF structures to be included. Twelve Cd analogues (CdIFs) have been reported by Tian et al.[2] and have been included here.

Table 1: ZIF structures in main, coarse-grained database

| Composition | DepNum/CCDC Code | Name | Topology | Included? | Details | Reference |
|---|---|---|---|---|---|---|
| $Zn(eIm)_2$ | 287181/MECWIB | ZIF-14 | ANA | y | - | [3] |
| $Cd(eIm)_2$ | 743555/GUPCOK | CdIF-6 | ANA | y | - | [2] |
| $Cd(nPrIm)_2$ | 743560/GUPDIF | CdIF-10 | ANA | y | - | [2] |
| $Cd(nBuIm)_2$ | 743561/GUPDOL | CdIF-11 | ANA | y | - | [2] |
| $Co(Im)_2$ | 228486/EQOCES01 | - | cag | y | - | [4] |
| $Zn(nIm)_2$ | 671070/GIZJOP | ZIF-62 | cag | y | - | [5] |
| $Co(Im)_2$ | 223195/NAFGOR | - | cag | y | - | [4] |
| $Zn(Im)_2$ | 602536/VEJYUF | ZIF-4 | cag | y | - | [6] |
| $Zn(Im)_2$ | 254158/VEJUF01 | - | cag | y | - | [7] |
| $Zn(Im)_{1.5}(mbIm)_{0.5}$ | 701064/QOSYAZ | TIF-4 | cag | y | - | [8] |
| $Zn(Im)_2$ | 212357/EQOCOC | - | coi | y | - | [9] |
| $Co(Im)_2$ | 1180335/IMZYCO | - | coi | y | - | [10] |
| $Zn(Im)_2$ | 671071/GITTEJ | ZIF-64 | crb(BCT) | y | - | [5] |
| $Fe(mIm)_2$ | 1208073/LODCUC | - | crb(BCT) | y | Disordered ligand - take avg. pos. | [11] |
| $Co(Im)_2$ | 223194/NAFGOR01 | - | crb(BCT) | y | - | [4] |
| $Zn(Im)_2$ | 602535/VEJYEP | ZIF-1 | crb(BCT) | y | - | [6] |
| $Zn(Im)_2$ | 254157/VEJYEP01 | - | crb(BCT) | y | - | [7] |
| $Zn_2(Im)_4$ | 602536/VEJYIT | ZIF-2 | crb(BCT) | y | - | [6] |
| $Zn(Im)_2$ | 254159/VEJYIT01 | - | crb(BCT) | y | - | [7] |
| $Pr(Im)_5$ | 609489/LEMVOP | - | crs | n | Octahedral Pr environment | [12] |
| $Zn(Im)_2$ | 254160/HIFVOI | - | dft | y | - | [7] |
| $Zn_2(Im)_4$ | 602537/VEJYOZ | ZIF-3 | dft | y | - | [6] |
| $Zn(4abIm)_2$ | 647002/MIHHOB | ZIF-23 | dia | y | - | [13] |
| $Cd_2(HIm)_3(Im)$ | 655703/VIGHID | - | dia | n | Octahedral Cd environment | [14] |
| $Fe(4abIm)_2$ | 153520/XASGON | - | dia | y | - | [15] |
| $Fe(biIm)_2$ | 1312697/ZIMMEN | - | dia | n | Octahedral Fe environment | [16] |
| $Cd(nim)_2$ | 743556/GUPCUQ | CdIF-7$\alpha$ | dia | y | - | [2] |
| $Cd(nim)_2$ | 736757/GUPBOJ | CdIF-7$\beta$ | dia | y | - | [2] |
| $Cd(PhIm)_2$ | 743562/GUPDUR | CdIF-12 | dia | y | - | [2] |
| $Hg(Im)_2$ | 205573/BAYPUN | - | dia-c | y | - | [17] |
| $Cd(Im)_2$ | 205574/BAYQAU | - | dia-c | y | - | [17] |
| $Cd(Im)_2$ | 213369/BAYQAU01 | - | dia-c | y | - | [9] |
| $LiB(mIm)_4$ | 699084/MOXKUG | BIF-2Li | dia-c-b | y | - | [18] |
| $CuB(mIm)_4$ | 703703/MUCLIG | BIF-2Cu | dia-c-b | y | - | [18] |
| $CuBH(Im)_3$ | 697962/MOXKEQ | BIF-6 | fes | n | Pyramidal Cu | [18] |
| $Zn(nIm)_{1.74}(mbIm)_{0.26}$ | 671083/GITVOV | ZIF-73 | frl | y | Disordered ligand | [5] |
| $Zn(nIm)_2$ | 671089/GITWIQ | ZIF-77 | frl | y | - | [5] |
| $Zn_3In_2(Im)_{12}$ | 602539/VEJZAM | ZIF-5 | gar | n | Octahedral In environment | [6] |
| $Zn(Im)_2$ | 602540/EQOCOC01 | ZIF-6 | GIS | y | - | [6] |
| $Zn(mbIm)(nIm)$ | 671085/GITVUB | ZIF-74 | GIS | y | - | [5] |
| $Zn(Im)_2$ | 671086/GITWAI | ZIF-75 | GIS | y | - | [6] |
| $Zn(Im)_2$ | 254162/HIFVUO | - | GIS | y | - | [7] |

Table 1: ZIF structures in main, coarse-grained database

| Composition | DepNum/CCDC Code | Name | Topology | Included? | Details | Reference |
|---|---|---|---|---|---|---|
| Zn(Im)(dmbIm) | 701066/QOSYIH | TIF-5Zn | GIS | y | - | [8] |
| Co(Im)(dmbIm) | 701065/QOSYED | TIF-5Co | GIS | y | - | [8] |
| Zn(bIm)(nIm) | 671075/GITTUZ | ZIF-68 | GME | y | - | [5] |
| Zn(cbIm)(nIm) | 671076/GITVAH | ZIF-69 | GME | y | Thermal disorder in ligand | [5] |
| $Zn(Im)_{1.13}(nIm)_{0.87}$ | 671078/GITVEL | ZIF-70 | GME | y | - | [5] |
| Zn(nbIm)(nIm) | 704995/YOZBIZ | ZIF-78 | GME | y | Thermal disorder in ligand | [19] |
| Zn(mbIm)(nIm) | 704997/YOZBOF | ZIF-79 | GME | y | - | [19] |
| Zn(dcIm)(nIm) | 704999/YOZBUL | ZIF-80 | GME | y | - | [19] |
| Zn(brbIm)(nIm) | 705001/YOZCAS | ZIF-81 | GME | y | - | [19] |
| Zn(cnIm)(nIm) | 705002/YOZCEW | ZIF-82 | GME | y | - | [19] |
| Cd(mIm)2 | 743554/GUPBOJ01 | CdIF-5 | ict | y | - | [2] |
| $Zn(dcIm)_2$ | 671082/GIZJUV | ZIF-72 | lcs | y | - | [5] |
| Zn(Im)(cbIm) | 671087/GITWEM | ZIF-76 | LTA | y | - | [5] |
| $Zn(pur)_2$ | 646999/MIHHAN | ZIF-20 | LTA | y | - | [13] |
| $Co(pur)_2$ | 647000/MIHHER | ZIF-21 | LTA | y | - | [13] |
| $Zn(5abIm)_2$ | 647001/MIHHIV | ZIF-22 | LTA | y | - | [13] |
| $Cd(Im)_2(bipy)$ | 294000/DAYVIJ | - | mab | n | Octahedral Cd environment | [20] |
| $In_5(Imdc)_{10}$ | 690432/EGEHOO | usf-ZMOF | med | y | - | [21] |
| $Zn_2(Im)_3(mIm)$ | 671067/GITSUY | ZIF-60 | MER | y | - | [5] |
| $Zn(Im)_2$ | 602544/VEJZIU | ZIF-10 | MER | y | - | [6] |
| $Cd(mIm)_2$ | 743552/GUPCAW | CdIF-2 | MER | y | - | [2] |
| $Cu(Im)_2$ | 159163/CUIMDZ03 | - | mog | y | - | [22] |
| $Fe_3(Im)_6$ | 1180191/IMIDFE | - | mog | n | Octahedral Fe environment | [23] |
| $Fe_3(Im)_6$ | 1180192/IMIDFE01 | - | mog | n | Octahedral Fe environment | [24] |
| $Mn_3(Im)_6$ | 1180193/IMIDZA | - | mog | n | Octahedral Mn environment | [25] |
| $Zn_{20}(cbIm)_{39}(OH)$ | 668215/NOFQEF | ZIF-100 | moz | n | Network modifier present | [26] |
| $Co(Im)_2$ | 149555/EQOBUH | - | neb | y | - | [9] |
| $Co(Im)_2$ | 212355/EQOCES | - | neb | y | - | [9] |
| $Co(Im)_2$ | 212356/EQOCIW | - | neb | y | - | [9] |
| $Co_5(Im)_{10}$ | 168798/AFIXAO | - | nog | y | - | [9] |
| $Co_5(Im)_{10}$ | 168799/AFIXES | - | nog | y | - | [27] |
| $Zn(Im)_2$ | 646539/HIFWAV | - | nog | y | - | [7] |
| Zn(Im)(mbIm) | 701063/QOSXUS | TIF-3 | pcb(ACO) | y | Different ligand - adapted cg procedure | [8] |
| $Zn(cbIm)_2$ | 668214/NOFQAB | ZIF-95 | poz | y | - | [26] |
| $Fe(mIm)_2$ | 1119170/CAGLIF | - | qtz | y | - | [28] |
| $Zn(dcIm)_2$ | 671080/GITVIP | ZIF-71 | RHO | y | - | [5] |
| $Zn_2(eIm)_4$ | 287182/MECWOH | - | RHO | y | - | [3] |
| $In(Imdc)_2$ | 294663/TEFWIL | rho-ZMOF | RHO | n | In not tetrahedral | [29] |
| $Zn(bIm)_2$ | 602545/VEJZOA | ZIF-11 | RHO | y | - | [6] |

Table 1: ZIF structures in main, coarse-grained database

| Composition | DepNum/CCDC Code | Name | Topology | Included? | Details | Reference |
|---|---|---|---|---|---|---|
| $Co(bIm)_2$ | 602546/VEJZUG | ZIF-12 | RHO | y | - | [6] |
| $Cd(eIm)_2$ | 743553/GUPCEA | CdIF-4 | RHO | y | - | [2] |
| $Cd(nIm)_2$ | 743559/GUPDEB | CdIF-9 | RHO | y | - | [2] |
| $Zn(lca)_2$ | 693596/WOJGEI | ZIF-90 | SOD | y | - | [30] |
| $Zn(bIm)_2$ | 211533/AKUGES | - | SOD | y | - | [31] |
| $Cu(Im)_2$ | 159161/CUIMDZ01 | - | SOD | y | - | [22] |
| $Co(nIm)_2$ | 671072/GITTIN | ZIF-65 | SOD | y | - | [5] |
| $Co(nIm)_2$ | 671073/GITTOT | ZIF-67 | SOD | y | - | [5] |
| $Zn(mIm)_2$ | 287180/MECWEX | - | SOD | y | - | [3] |
| $Zn(Im\text{-}d5)_2$ | 652032/OFERUN | ZIF-8(D) | SOD | n | Same as ZIF-8(H) | [32] |
| $In(Imdc)_2$ | 294664/TEFWOR | sod-ZMOF | SOD | n | In not tetrahedral | [29] |
| $Co(bIm)_2$ | 602543/VEJZEQ | ZIF-9 | SOD | y | - | [6] |
| $Zn(bIm)_2$ | 602541/VELVIS | ZIF-7 | SOD | y | - | [6] |
| $Zn(mIm)_2$ | 602542/VELVOY | ZIF-8 | SOD | y | - | [6] |
| $Cd(mIm)_2$ | 743551/GUPBUP | CdIF-1 | SOD | y | - | [2] |
| $Cd(nIm)_2$ | 743558/GUPCUQ01 | CdIF-8 | SOD | y | - | [2] |
| $LiB(mIm)_2$ | 703704/MUCLOM | BIF-3Li | SOD-b | y | - | [18] |
| $CuB(mIm)_2$ | 697959/MOXJOZ | BIF-3Cu | SOD-b | y | - | [18] |
| $CuBH(eIm)_3$ | 697964/MOXKOA | BIF-8 | srs-c-b | n | Pyramidal Cu | [18] |
| $CuBH(mIm)_3$ | 697963/MOXKIU | BIF-7 | ths-c-b | n | Not tetrahedral | [18] |
| $Cd(mim)_2$ | 736757/GUPBOJ | CdIF-3 | yqt1 | n | Disordered ligand | [2] |
| $Zn(dmbIm)_2$ | 682400/QOGPIM | TIF-1Zn | zea | y | Occupational disorder in ligand | [33] |
| $Zn(Im)_{1.10}(mbIm)_{0.9}$ | 701062/QOSXOM | TIF-2 | zeb | y | - | [8] |
| $Zn(Im)_2$ | 254161/HICGEG | - | zec | y | - | [7] |
| $Zn(Im)(mIm)$ | 671069/GITTAF | ZIF-61 | zni | y | - | [5] |
| $Zn(Im)_2$ | 1180194/IMIDZB | - | zni | y | - | [25] |
| $Co(Im)_2$ | 212358/IMZYCO01 | - | zni | y | - | [9] |
| $LiB(Im)_2$ | 693499/MOXJEP | BIF-1Li | zni-b | y | - | [18] |
| $CuB(Im)_2$ | 693500/MOXJIT | BIF-1Cu | zni-b | y | - | [18] |
| $CuCu(Im)_3$ | 227576/BETHUE | - | - | n | Linear Cu | [34] |
| $CuCu[B(bIm)_4]_2$ | 697960/MOXJUF | BIF-4 | - | n | Ligand disorder | [18] |
| $Cu3I[B(bIm)_4]_2$ | 697961/MOXKAM | BIF-5 | - | n | Network modifier | [18] |

All of the compact polymorphic forms of silica have been included in the dataset. Structures have been downloaded from the ICSD and the American Mineralogist Crystal Structure Database (AMCSD).[35]

Table 2: Silica structures included in the database

| Mineral | ICSD (AMCSD) Identifier | Reference |
|---|---|---|
| Coesite | 156195 | [36] |
| Cristobalite-high | (0017665) | [37] |
| Cristobalite-low | (0017659) | [38] |
| Tridymite-high | (0020733) | [39] |
| Tridymite-low | (0009625) | [40] |
| Keatite | 34889 | [41] |
| Moganite | 67669 | [42] |
| Quartz-high* | - | [43] |
| Quartz-low | 173227 | [44] |
| Stishovite | not inlcuded | Octahedral Si environment |

*Crystallographic information used corresponds to the synthetic crystal (590°C), O general dataset, taken from Table 1 of this reference.

All structures for the experimentally realised Zeolites and related microporous materials have been taken from the IZA-database. For each framework type, the cif files provided have been included in the database.

Table 3: Aluminophosphate structures included in the database

| Mineral | Topology | DepNum | Description | Reference |
|---|---|---|---|---|
| Li-A(BW) | ABW | IZA-SC | n - $H_2O$ network-modifier | [47] |
| ACP-1 | ACO | 661416/BEFNIL | y | [48] |
| AlPO-18(calcined) | AEI | IZA-SC | y | [49] |
| AlPO-11(calcined) | AEL | IZA-SC | y | [50] |
| MAPO-11 | AEL | IZA-SC | y | [51] |
| AlPO-53(A) | AEN | IZA-SC | n - penta-coordinate Al | [52] |
| AlPO-8 | AET | 1635179 | y | [53] |
| Afghanite | AFG | IZA-SC | y | [54] |
| AlPO-5 | AFI | 1651199/(91673) | y | [55] |
| AlPO-14(calcined) | AFN | IZA-SC | n - not fully connected | [56] |
| AlPO-41(calcined) | AFO | IZA-SC | y | [57] |
| SAPO-40 | AFR | IZA-SC | y | [58] |
| MAPSO-46 | AFS | IZA-SC | y | [59] |
| AlPO-52(calcined) | AFT | IZA-SC | y | [60] |
| AlPO-57 | AFV | IZA-SC | y | [61] |
| SAPO-56 | AFX | IZA-SC | y | [62] |
| CoAPO-50 | AFY | IZA-SC | y | [59] |
| AlPO-H2 | AHT | IZA-SC | n - Octahedral Al environment | [63] |
| Analcime | ANA | IZA-SC | y | [64] |
| AlPO-C(hydrated) | APC | IZA-SC | n - Octahedral Al environment with $H_2O$ network modifiers | [65] |
| AlPO-C(dehydrated) | APC | IZA-SC | y | [66] |
| AlPO-D | APD | IZA-SC | y | [66] |
| Octadecasil | AST | IZA-SC | y | [67] |
| AlPO-16 | AST | IZA-SC | y | [68] |
| ASU-7 | ASV | IZA-SC | y | [69] |
| MAPO-39 | ATN | IZA-SC | n - Disordered | [70] |
| SAPO-31 | ATO | IZA-SC | y | [71] |
| MAPO-36(calcined) | ATS | IZA-SC | y | [72] |
| B-SSZ-55 | ATS | IZA-SC | y | [73] |
| AlPO-12 | ATT | IZA-SC | y | [74] |
| AlPO-25 | ATV | IZA-SC | y | [75] |
| AlPO-78 | AVE | IZA-SC | y | [76] |
| AlPO-59 | AVL | IZA-SC | n - disordered | [61] |
| AlPO-21 | AWO | IZA-SC | n - 5 coordinate Al | [75] |
| AlPO-22 | AWW | IZA-SC | n - 5 coordinate Al | [77] |
| Mg-BCTT | BCT | IZA-SC | y | [78] |
| FOS-5 | BEC | IZA-SC | y | [79] |
| ITQ-17 | BEC | IZA-SC | y | [80] |
| Bikitaite | BIC | IZA-SC | n - $H_2O$ network-modifier | [81] |
| UCSB-15GaGe | BOF | IZA-SC | y | [82] |
| Boggsite | BOG | IZA-SC | y | [83] |
| Be-10 | BOZ | IZA-SC | y | [84] |
| BePhosphate-H | BPH | IZA-SC | y | [85] |
| Brewsterite | BRE | IZA-SC | y | [86] |
| UCSB-7 | BSV | IZA-SC | y | [82] |
| Cancrinite | CAN | IZA-SC | y | [87] |
| Tiptopite | CAN | IZA-SC | y | [88] |

Table 3: Aluminophosphate structures included in the database

| Mineral | Topology | DepNum | Description | Reference |
|---|---|---|---|---|
| Cs-aluminosilicate | CAS | IZA-SC | y | [89] |
| UZM-25(calcined) | CDO | IZA-SC | y | [90] |
| CIT-5 | CFI | IZA-SC | y | [91] |
| CoGaPO-5 | CGF | IZA-SC | y | [92] |
| CoGaPO-6 | CGS | IZA-SC | y | [93] |
| Chabazite | CHA | IZA-SC | y | [94] |
| SAPO-47 | CHA | IZA-SC | y | [95] |
| CIT-1 | CON | IZA-SC | n - cif file missing some O positions | [96] |
| CIT-7 | CSV | IZA-SC | y | [97] |
| Na-ZnPO | CZP | IZA-SC | y - used $P6_522$ as less disordered | [98] |
| Dachiardite | DAC | IZA-SC | y | [99] |
| Deca-dodecasil-3R | DDR | IZA-SC | y | [100] |
| DAF-1 | DFO | IZA-SC | y | [101] |
| UiO-20 | DFT | IZA-SC | y | [102] |
| Dodecasil-1H | DOH | IZA-SC | y | [103] |
| UTD-1F | DON | IZA-SC | y | [104] |
| Bellbergite | EAB | IZA-SC | y | [105] |
| TMA-E | EAB | IZA-SC | y | [106] |
| Edingtonite | EDI | IZA-SC | y | [107] |
| K-F | EDI | IZA-SC | y | [108] |
| SSZ-45 | EEI | IZA-SC | y | [109] |
| EMC-2(calcined) | EMT | IZA-SC | y | [110] |
| TNU-7 | EON | IZA-SC | y | [111] |
| Epistilbite | EPI | IZA-SC | y | [112] |
| Erionite | ERI | IZA-SC | y | [113] |
| AlPO-17 | ERI | IZA-SC | y | [114] |
| ERS-7 | ESV | IZA-SC | y | [115] |
| EU-12 | ETL | IZA-SC | y | [116] |
| ECR-34 | ETR | IZA-SC | y | [117] |
| EU-1 | EUO | IZA-SC | y | [118] |
| ZSM-50 | EUO | IZA-SC | y | [119] |
| EMM-26 | EWS | IZA-SC | y | [120] |
| EMM-3 | EZT | IZA-SC | y | [121] |
| Farneseite | FAR | IZA-SC | y | [122] |
| Faujasite | FAU | IZA-SC | y | [123] |
| Na-X | FAU | IZA-SC | y | [124] |
| US-Y | FAU | IZA-SC | n - used siliceuous form | [125] |
| Na-Y(siliceuous) | FAU | IZA-SC | y | [126] |
| Li-LSX | FAU | IZA-SC | y | [127] |
| Ferrierite | FER | IZA-SC | y | [127] |
| Ferrierite(siliceous) | FER | IZA-SC | y | [128] |
| Franzinite | FRA | IZA-SC | y | [129] |
| Gismondine | GIS | IZA-SC | y | [130] |
| Amicite | GIS | IZA-SC | y | [131] |
| Garronite | GIS | IZA-SC | y | [131] |
| Gobbinsite | GIS | IZA-SC | y | [132] |
| MAPO-43 | GIS | IZA-SC | y | [133] |
| Na-P1 | GIS | IZA-SC | y | [134] |
| Giuseppettite | GIU | IZA-SC | y | [135] |
| Gmelinite | GME | IZA-SC | y | [136] |

Table 3: Aluminophosphate structures included in the database

| Mineral | Topology | DepNum | Description | Reference |
|---|---|---|---|---|
| GUS-1 | GON | IZA-SC | y | [137] |
| Goosecreekite | GOO | IZA-SC | y | [138] |
| Heulandite | HEU | IZA-SC | y | [139] |
| Clinoptilolite | HEU | IZA-SC | y | [140] |
| ITQ-51 | IFO | IZA-SC | y | [141] |
| ITQ-4(calcined) | IFR | IZA-SC | y | [142] |
| ITQ-52(calcined) | IFW | IZA-SC | y | [143] |
| SSZ-87(as-made) | IFW | IZA-SC | n - use (simpler) calcined form | [144] |
| SSZ-87(as-synthesised) | IFW | IZA-SC | n - use (simpler) calcined form | [144] |
| SSZ-87(calcined) | IFW | IZA-SC | y | [144] |
| ITQ-50(calcined) | IFY | IZA-SC | y | [145] |
| ITQ-32 | IHW | IZA-SC | y | [146] |
| IM-5(calcined) | IMF | IZA-SC | y | [147] |
| ITQ-49(calcined) | IRN | IZA-SC | y | [148] |
| ITQ-44 | IRR | IZA-SC | y | [149] |
| ITQ-7(calcined) | ISV | IZA-SC | y | [150] |
| ITQ-3(calcined) | ITE | IZA-SC | y | [151] |
| ITQ-38(calcined) | ITG | IZA-SC | y | [152] |
| ITQ-13 | ITH | IZA-SC | y | [153] |
| ITQ-34 | ITR | IZA-SC | y | [154] |
| ITQ-33 | ITT | IZA-SC | n - disordered | [155] |
| ITQ-12(calcined) | ITW | IZA-SC | y | [156] |
| ITQ-24 | IWR | IZA-SC | y | [157] |
| ITQ-26 | IWS | IZA-SC | y | [158] |
| ITQ-27 | IWV | IZA-SC | y | [159] |
| ITQ-22 | IWW | IZA-SC | y | [160] |
| Nepheline Hydrate | JBW | IZA-SC | n - not fully connected | [161] |
| JU92-300 | JNT | IZA-SC | y | [162] |
| LSJ-10 | JOZ | IZA-SC | y | [163] |
| CoAPO-CJ40 | JRY | IZA-SC | n - disordered | [164] |
| CoAPO-CJ69 | JSN | IZA-SC | y | [165] |
| JU-64 | JSR | IZA-SC | y | [166] |
| GaGeO-CJ63 | JST | IZA-SC | y | [167] |
| CoAPO-CJ62 | JSW | IZA-SC | y | [168] |
| (Cs,K)-ZK-5 | KFI | IZA-SC | y | [169] |
| Laumontite | LAU | IZA-SC | y | [170] |
| Leonhardite | LAU | IZA-SC | y | [171] |
| CoGaPO | LAU | IZA-SC | y | No reference given |
| Levyne | LEV | IZA-SC | y | [172] |
| Nu-3 | LEV | IZA-SC | y | [173] |
| Liottite | LIO | IZA-SC | y | [174] |
| Losod | LOS | IZA-SC | y | [175] |
| Lovdarite | LOV | IZA-SC | y | [176] |
| Linde-Type-A(hydrated) | LTA | IZA-SC | n - (used dehydrated form) | [177] |
| Linde-Type-A(dehydrated) | LTA | IZA-SC | y | [178] |
| LZ-135 | LTF | IZA-SC | y | [179] |
| Linde-Type-J | LTJ | IZA-SC | y | [180] |

Table 3: Aluminophosphate structures included in the database

| Mineral | Topology | DepNum | Description | Reference |
|---|---|---|---|---|
| Perlialite | LTL | IZA-SC | y | [181] |
| Linde-Type-L | LTL | IZA-SC | y | [182] |
| NaZ-21 | LTN | IZA-SC | y | [183] |
| Marinellite | MAR | IZA-SC | n - not fully connected | [184] |
| Mazzite | MAZ | IZA-SC | y | [185] |
| ZSM-18 | MEI | IZA-SC | y | [186] |
| ZSM-11(calcined) | MEL | IZA-SC | y | [187] |
| ZSM-11(siliceuous) | MEL | IZA-SC | y | [188] |
| Melanophlogite | MEP | IZA-SC | y | [189] |
| Merlinoite | MER | IZA-SC | y | [190] |
| BaClAPO | MER | IZA-SC | y | [191] |
| ZSM-5 | MFI | IZA-SC | n - use calcined instead | [192] |
| ZSM-5(calcined) | MFI | IZA-SC | y | [193] |
| ZSM-57 | MFS | IZA-SC | y | [194] |
| Montesommaite | MON | IZA-SC | y | [195] |
| Mordenite | MOR | IZA-SC | y | [177] |
| Maricopaite | MOR | IZA-SC | n - not fully connected | [196] |
| ZSM-10 | MOZ | IZA-SC | y | [197] |
| ZSM-43 | MRT | IZA-SC | y | [198] |
| MCM-68 | MSE | IZA-SC | y | [199] |
| MCM-61 | MSO | IZA-SC | n - unrecognised space-group | [200] |
| MCM-35 | MTF | IZA-SC | y | [201] |
| Dodecasil-3C | MTN | IZA-SC | y | [202] |
| ZSM-23 | MTT | IZA-SC | y | [203] |
| ZSM-12(calcined) | MTW | IZA-SC | y | [204] |
| MCM-70 | MVY | IZA-SC | y | [205] |
| ZSM-25 | MWF | IZA-SC | y | [206] |
| ITQ-1(calcined) | MWW | IZA-SC | y | [207] |
| Nabesite | NAB | IZA-SC | y | [208] |
| Natrolite | NAT | IZA-SC | y | [209] |
| Mesolite | NAT | IZA-SC | y | [210] |
| Scolecite | NAT | IZA-SC | y | [211] |
| Nu-87 | NES | IZA-SC | y | [212] |
| Nonasil | NON | IZA-SC | y | [213] |
| NPO-1 | NPO | IZA-SC | y | [214] |
| ONPO-2 | NPT | IZA-SC | y | [215] |
| Nu-6(2) | NSI | IZA-SC | y | [216] |
| OSB-2 | OBW | IZA-SC | y | [217] |
| Offretite | OFF | IZA-SC | y | [218] |
| COK-14 | OKO | IZA-SC | y | [219] |
| UiO-6(calcined) | OSI | IZA-SC | y | [220] |
| OSB-1 | OSO | IZA-SC | y | [217] |
| UiO-28 | OWE | IZA-SC | y | [221] |
| Paulingite | PAU | IZA-SC | y | [222] |
| IPC-4 | PCR | IZA-SC | y | [223] |
| Phillipsite | PHI | IZA-SC | y | [224] |
| Harmotome | PHI | IZA-SC | y | [224] |
| IST-1 | PON | IZA-SC | y | [225] |
| PST-13 | POR | IZA-SC | y | [226] |
| PST-14 | POR | IZA-SC | y | [226] |
| PUK-16 | POS | IZA-SC | y | [227] |

Table 3: Aluminophosphate structures included in the database

| Mineral | Topology | DepNum | Description | Reference |
|---|---|---|---|---|
| PST-6 | PSI | IZA-SC | y | [228] |
| PUK-9 | PUN | IZA-SC | y | [229] |
| PST-29 | PWN | IZA-SC | y | [230] |
| PST-21 | PWO | IZA-SC | y | [231] |
| PST-22 | PWW | IZA-SC | y | [231] |
| Pahasapaite | RHO | IZA-SC | n - not fully connected | [232] |
| Rho(hydrated) | RHO | IZA-SC | n - use deuterated form | [233] |
| Rho(deuterated) | RHO | IZA-SC | y | [234] |
| RUB-41 | RRO | IZA-SC | y | [235] |
| RUB-17 | RSN | IZA-SC | y | [236] |
| RUB-3 | RTE | IZA-SC | y | [237] |
| RUB-13 | RTH | IZA-SC | y | [238] |
| RUB-10 | RUT | IZA-SC | y | [239] |
| RUB-24 | RWR | IZA-SC | y | [240] |
| UCR-20 | RWY | IZA-SC | y | [241] |
| STA-15 | SAF | IZA-SC | y | [242] |
| STA-1 | SAO | IZA-SC | y | [243] |
| STA-6 | SAS | IZA-SC | y | [244] |
| STA-2 | SAT | IZA-SC | y | [245] |
| STA-7 | SAV | IZA-SC | y | [246] |
| UCSB-8Co | SBE | IZA-SC | y | [247] |
| UCSB-9 | SBN | IZA-SC | y | [248] |
| UCSB-6GaCo | SBS | IZA-SC | y | [247] |
| UCSB-10GaZn | SBT | IZA-SC | y | [247] |
| SSZ-82 | SEW | IZA-SC | y | [249] |
| SSZ-48(calcined) | SFE | IZA-SC | y | [250] |
| SSZ-44(calcined) | SFF | IZA-SC | y | [251] |
| SSZ-58 | SFG | IZA-SC | y | [252] |
| SSZ-53 | SFH | IZA-SC | y | [253] |
| SSZ-59 | SFN | IZA-SC | y | [253] |
| SSZ-51 | SFO | IZA-SC | y | [254] |
| SSZ-56 | SFS | IZA-SC | y | [255] |
| SSZ-52 | SFW | IZA-SC | y | [256] |
| Sigma-2 | SGT | IZA-SC | y | [257] |
| SIZ-7 | SIV | IZA-SC | y | [258] |
| Sodalite | SOD | IZA-SC | y | [259] |
| Bicchulite | SOD | IZA-SC | y | [260] |
| Tugtupite | SOD | IZA-SC | y | [261] |
| SU-15 | SOF | IZA-SC | y | [262] |
| SCM-14 | SOR | IZA-SC | y | [263] |
| SU-16 | SOS | IZA-SC | y | [264] |
| SCM-15 | SOV | IZA-SC | y | [265] |
| SSZ-65 | SSF | IZA-SC | y | [266] |
| SSZ-60 | SSY | IZA-SC | y | [267] |
| SSZ-35(calcined) | STF | IZA-SC | y | [251] |
| Stilbite | STI | IZA-SC | y | [268] |
| Stellerite | STI | IZA-SC | y | [269] |
| Barrerite | STI | IZA-SC | y | [270] |
| SSZ-23 | STT | IZA-SC | y | [271] |
| SU-32 | STW | IZA-SC | y | [262] |
| SSZ-77 | SVV | IZA-SC | y | [272] |
| STA-20 | SWY | IZA-SC | y | [273] |

Table 3: Aluminophosphate structures included in the database

| Mineral | Topology | DepNum | Description | Reference |
|---|---|---|---|---|
| SUZ-4 | SZR | IZA-SC | y | [274] |
| Terranovaite | TER | IZA-SC | y | [275] |
| Thomsonite | THO | IZA-SC | y | [276] |
| Tounkite-like | TOL | IZA-SC | y | [277] |
| ZSM-22 (Theta-1) | TON | IZA-SC | y | [278] |
| Tschörtnerite | TSC | IZA-SC | y | [279] |
| TNU-9 | TUN | IZA-SC | y | [280] |
| Mu-18 | UEI | IZA-SC | y | [281] |
| UZM-5 | UFI | IZA-SC | y | [282] |
| IM-16 | UOS | IZA-SC | y | [283] |
| IM-17 | UOV | IZA-SC | y | [284] |
| IM-10 | UOZ | IZA-SC | y | [285] |
| IM-6 | USI | IZA-SC | y | [286] |
| IM-12 | UTL | IZA-SC | y | [287] |
| IM-20 | UWY | IZA-SC | y | [288] |
| VPI-8 | VET | IZA-SC | y | [289] |
| VPI-5 | VFI | IZA-SC | y | [290] |
| VPI-9 | VNI | IZA-SC | n - not fully connected | [290] |
| VPI-7 | VSV | IZA-SC | y | [291] |
| Weinebeneite | WEI | IZA-SC | y | [292] |
| YNU-5 | YFI | IZA-SC | y | [293] |
| Yugawaralite | YUG | IZA-SC | y | [294] |
| ZAPO-M1 | ZON | IZA-SC | y | [295] |

All seventeen experimentally realised ice forms have been considered. Entirely ordered and entirely disordered structures were included in the dataset.

Table 4: Ice structures included in the database

| Mineral | Topology | ICSD Identifier | Included? | H-Ordering | Reference |
|---|---|---|---|---|---|
| Ice-Ic | dia | 64775 | y | Disordered | [296] |
| Ice-Ih | lon | 64776 | y | Disordered | [297] |
| Ice-II | ict | 15907 | y | Ordered | [298] |
| Ice-III | kea | 64771 | y | Disordered | [299] |
| Ice-IV | icf | 201179 | y | Disordered | [300] |
| Ice-V | icv | 14318 | y | Disordered | [301] |
| Ice-VI | edi | - | y | Disordered | [302] |
| Ice-VII | itv | 31868 | y | Disordered | [303] |
| Ice-VIII | dia | 1613533 | y | Ordered | [304] |
| Ice-IX | kea | 1614920 | y | Ordered | [305] |
| Ice-X | itv | - | n | Symmetric | |
| Ice-XI | lon | - | y | Ordered | [306] |
| Ice-XII | itv | - | y | Disordered | [307] |
| Ice-XIII | icv | 1672152 | y | Ordered | [308] |
| Ice-XIV | itv | 1672153 | n | Partial ordering | [308] |
| Ice-XV | edi | 1682436 | y | Ordered | [309] |
| Ice-XVI | mtn | 1442023 | y | Disordered | [310] |
| Ice-XVII | unj | - | y | Disordered | [311] |

The cyanide structures included are taken from the paper “Exploiting High Pressures to Generate Porosity, Polymorphism, And Lattice Expansion in the Nonporous Molecular Framework $Zn(CN)_2$”, Saul H. Lapidus, Gregory J. Halder, Peter J. Chupas, Karena W. Chapman.

Table 5: Cyanide structures included in the database

| Formula | Phase | Description | Included? | Reference |
|---|---|---|---|---|
| $Zn(CN)_2$ | I (dia) | interpenetrated diamondoid | y | [312] |
| $Zn(CN)_2$ | II (dia) | distorted interpenetrated diamondoid | y | [312] |
| $Zn(CN)_2$ | dia | diamondoid | y | [312] |
| $Zn(CN)_2$ | lon | lonsdaleite | y | [312] |
| $Zn(CN)_2$ | pyr | pyrite | n - Octahedral Zn environment | [312] |

Table 6: Clathrate structures included in the database

| Name | Database Identifier | Clathrate Type | Included? | Reference |
|---|---|---|---|---|
| Acetylene clathrate | 247246/NAHCIJ | Type-I | y | [313] |
| $HPF_6$-7.67$H_2O$ | 1706893* | Type-I | y | [314] |
| Methane clathrate | 247245/NAHCEF | Type-I | y | [313] |
| Ethylene Oxide Hydrate | 1150521/ETHYLO | Type-I | y | [315] |
| Propane clathrate | 247247/NAHCOP | Type-II | y | [313] |
| Methane-propane clathrate | 247248/NAHCUV | Type-II | y | [313] |
| $XeCCl_4$-136$D_2O$ | 1194852/KELKUH | Type-II | y | [316] |
| Methane-adamantane clathrate | 247249/NAHDAC | Type-H | y | [313] |

*In the absence of a CCDC Identifier, an ICSD Identifier is provided.

Table 7: Inorganic structures included in the database

| Name | ICSD Identifier | Included? | Reference |
|---|---|---|---|
| alpha-$BeCl_2$ | 92583 | y | [317] |
| beta-$BeCl_2$ | 92586 | y | [317] |
| alpha-$ZnCl_2$ | 26154 | y | [318] |
| beta-$ZnCl_2$ | 26153 | y | [318] |
| gamma-$ZnCl_2$ | 26152 | y | [318] |
| $SiS_2$ | 291208 | y | [319] |
| $LiCo(CO)_2$ | 1712939* | y | [320] |

* Downloaded from the "Pauling File Multinaries Edition - 2012" database.[321]

# Bibliography


[1] Phan, A.; Doonan, C. J.; Uribe-Romo, F. J.; Knobler, C. B.; O'Keeffe, M.; Yaghi, O. M. *Acc. Chem. Res.* **2010,** *43,* 58–67.

[2] Tian, Y. Q.; Yao, S. Y.; Gu, D.; Cui, K. H.; Guo, D. W.; Zhang, G.; Chen, Z. X.; Zhao, D. Y. *Chem. - A Eur. J.* **2010,** *16,* 1137–1141.

[3] Huang, X.-C.; Lin, Y.-Y.; Zhang, J.-P.; Chen, X.-M. *Angew. Chemie Int. Ed.* **2006,** *45,* 1557–1559.

[4] Tian, Y. Q.; Chen, Z. X.; Weng, L. H.; Guo, H. B.; Gao, S.; Zhao, D. Y. *Inorg. Chem.* **2004,** *43,* 4631–4635.

[5] Banerjee, R.; Phan, A.; Wang, B.; Knobler, C.; Furukawa, H.; O'Keeffe, M.; Yaghi, O. M. *Science (80-. ).* **2008,** *319,* 939–943.

[6] Park, K. S.; Ni, Z.; Côté, A. P.; Choi, J. Y.; Huang, R.; Uribe-Romo, F. J.; Chae, H. K.; O'Keeffe, M.; Yaghi, O. M. *Proc. Natl. Acad. Sci. U. S. A.* **2006,** *103,* 10186–10191.

[7] Tian, Y. Q.; Zhao, Y. M.; Chen, Z. X.; Zhang, G. N.; Weng, L. H.; Zhao, D. Y. *Chem. - A Eur. J.* **2007,** *13,* 4146–4154.

[8] Wu, T.; Bu, X.; Zhang, J.; Feng, P. *Chem. Mater.* **2008,** *20,* 7377–7382.

[9] Tian, Y. Q.; Cai, C. X.; Ren, X. M.; Duan, C. Y.; Xu, Y.; Gao, S.; You, X. Z. *Chem. - A Eur. J.* **2003,** *9,* 5673–5685.

[10] Sturm, M.; Brandl, F.; Engel, D.; Hoppe, W. *Acta Crystallogr. Sect. B Struct. Crystallogr. Cryst. Chem.* **1975,** *31,* 2369–2378.

[11] Rettig, S. J.; Storr, A.; Summers, D. A.; Thompson, R. C.; Trotter, J. *Can. J. Chem.* **1999,** *77,* 425–433.

[12] Müller-Buschbaum, K. *Zeitschrift für Naturforsch. B* **2015,** *61,* 792–798.

[13] Hayashi, H.; Côté, A. P.; Furukawa, H.; O'Keeffe, M.; Yaghi, O. M. *Nat. Mater.* **2007,** *6,* 501–506.

[14] Fu, Y. M.; Zhao, Y. H.; Lan, Y. Q.; Wang, Y.; Qiu, Y. Q.; Shao, K. Z.; Su, Z. M. *Inorg. Chem. Commun.* **2007,** *10,* 720–723.

[15] Rcttig, S. J.; Sânchez, V.; Storr, A.; Thompson, R. C.; Trotter, J. *J. Chem. Soc. Dalt. Trans.* **2000,** *21,* 3931–3937.

[16] Lorente, M. A. M.; Dahan, F.; Sanakis, Y.; Petrouleas, V.; Bousseksou, A.; Tuchagues, J. P. *Inorg. Chem.* **1995,** *34,* 5346–5357.

[17] Masciocchi, N.; Ardizzoia, G. A.; Brenna, S.; Castelli, F.; Galli, S.; Maspero, A.; Sironi, A. *Chem. Commun.* **2003,** *3,* 2018–2019.

[18] Zhang, J.; Wu, T.; Zhou, C.; Chen, S.; Feng, P.; Bu, X. *Angew. Chemie - Int. Ed.* **2009,** *48,* 2542–2545.

[19] Banerjee, R.; Furukawa, H.; Britt, D.; Knobler, C.; O'Keeffe, M.; Yaghi, O. M. *J. Am. Chem. Soc.* **2009,** *131,* 3875–3877.

[20] Han, J. Y.; Fang, J.; Chang, H. Y.; Dong, Y.; Liang, S. *Acta Crystallogr. Sect. E Struct. Reports Online* **2005,** *61,*.

[21] Liu, Y.; Kravtsov, V. C.; Eddaoudi, M. *Angew. Chemie - Int. Ed.* **2008,** *47,* 8446–8449.

[22] Masciocchi, N.; Bruni, S.; Cariati, E.; Cariati, F.; Galli, S.; Sironi, A. *Inorg. Chem.* **2001,** *40,* 5897–5905.

[23] Lehnert, R.; Seel, F. *Z. anorg. allg. Chem.* **1978,** *444,* 91–96.

[24] Rettig, S. J.; Storr, A.; Summers, D. A.; Thompson, R. C.; Trotter, J. *J. Am. Chem. Soc.* **1997,** *119,* 8675–8680.

[25] Lehnert, R.; Seel, F. *ZAAC â J. Inorg. Gen. Chem.* **1980,** *464,* 187–194.

[26] Wang, B.; Côté, A. P.; Furukawa, H.; O'Keeffe, M.; Yaghi, O. M. *Nature* **2008,** *453,* 207–211.

[27] Tian, Y.-q.; Cai, C.-x.; Ji, Y.; You, X.-z.; Peng, S.-m.; Lee, G.-h. *Angew. Chem.* **2002,** *114,* 1384–1386.

[28] Spek, A. L.; Duisenberg, A. J. M.; Feiters, M. C. *Acta Crystallogr. Sect. C Cryst. Struct. Commun.* **1983,** *39,* 1212–1214.

[29] Liu, Y.; Kravtsov, V. C.; Larsen, R.; Eddaoudi, M. *Chem. Commun.* **2006,** *14,* 1488–1490.

[30] Morris, W.; Doonan, C. J.; Furukawa, H.; Banerjee, R.; Yaghi, O. M. *J. Am. Chem. Soc.* **2008,** *130,* 12626–12627.

[31] Huang, X.; Zhang, J.; Chen, X. *Chinese Sci. Bull.* **2003,** *48,* 1531–1534.

[32] Wu, H.; Zhou, W.; Yildirim, T. *J. Am. Chem. Soc.* **2007,** *129,* 5314–5315.

[33] Wu, T.; Bu, X.; Liu, R.; Lin, Z.; Zhang, J.; Feng, P. *Chem. - A Eur. J.* **2008,** *14,* 7771–7773.

[34] Huang, X. C.; Zhang, J. P.; Lin, Y. Y.; Yu, X. L.; Chen, X. M. *Chem. Commun.* **2004,** *10,* 1100–1101.

[35] Downs, R. T.; Hall-Wallace, M. *Am. Mineral.* **2003,** *88,* 247–250.

[36] Ikuta, D.; Kawame, N.; Banno, S.; Hirajima, T.; Ito, K.; Rakovan, J. F.; Downs, R. T.; Tamada, O. *Am. Mineral.* **2007,** *92,* 57–63.

[37] Wyckoff, R. *Zeitschrift fur Krist.* **1925,** *62,* 189–200.

[38] Nieuwenkamp, W. *Zeitschrift fur Krist.* **1935,** *92,* 82–88.

[39] Dollase, W. A. *Acta Crystallogr.* **1967,** *23,* 617–623.

[40] Konnert, J. H.; Appleman, D. E. *Acta Crystallogr. Sect. B Struct. Crystallogr. Cryst. Chem.* **1978,** *34,* 391–403.

[41] Shropshire, J.; Keat, P. P.; Vaughan, P. A. *Zeitschrift für Krist.* **1959,** *112,* 409–413.

[42] Miehe, G.; Graetsch, H. *Eur. J. Mineral.* **1992,** *4,* 693–706.

[43] Wright, A. F.; Lehmann, M. S. *J. Solid State Chem.* **1981,** *36,* 371–380.

[44] Nikitin, A. N.; Markova, G. V.; Balagurov, A. M.; Vasin, R. N.; Alekseeva, O. V. *Crystallogr. Reports* **2007,** *52,* 428–435.

[45] Grazulis, S.; Chateigner, D.; Downs, R. T.; Yokochi, A. F. T.; Quiros, M.; Lutterotti, L.; Manakova, E.; Butkus, J.; Moeck, P.; Le Bail, A. *J. Appl. Crystallogr.* **2009,** *42,* 726–729.

[46] Merkys, A.; Vaitkus, A.; Butkus, J.; Okulic-Kazarinas, M.; Kairys, V.; Grazulis, S. *J. Appl. Crystallogr.* **2016,** *49,* 292–301.

[47] Andersen, E. K.; Ploug-Sørensen, G. *Zeitschrift für Krist. - Cryst. Mater.* **1986,** *176,*.

[48] Feng, P.; Bu, X.; Stucky, G. D. *Nature* **1997,** *388,* 735–741.

[49] Simmen, A.; McCusker, L. B.; Baerlocher, C.; Meier, W. M. *Zeolites* **1991,** *11,* 654–661.

[50] Richardson, J. W.; Pluth, J. J.; Smith, J. V. *Acta Crystallogr. Sect. B* **1988,** *44,* 367–373.

[51] Pluth, J. J.; Smith, J. V.; Richardson, J. W. *J. Phys. Chem.* **1988,** *92,* 2734–2738.

[52] Kirchner, R. M.; Grosse-Kunstleve, R. W.; Pluth, J. J.; Wilson, S. T.; Broach, R. W.; Smith, J. V. *Microporous Mesoporous Mater.* **2000,** *39,* 319–332.

[53] Poojary, M.; Clearfield, A. *Mater. Chem. Phys.* **1993,** *35,* 301–304.

[54] Pobedimskaya, E. A.; Rastsvetaeva, R. K.; Terent'eva, L. E.; Sapozhnikov, A. N. *Dokl. Akad. Nauk SSSR* **1991,** *320,* 882–6 [Crystallogr.].

[55] Klap, G. J.; Van Koningsveld, H.; Graafsma, H.; Schreurs, A. M. *Microporous Mesoporous Mater.* **2000,** *38,* 403–412.

[56] Broach, R. W.; Wilson, S. T.; Kirchner, R. M. Synthesis and structures of as-synthesized and calcined AlPO4-14 revealing a three-dimensional channel system with 8-ring pores.. In *Proc. Int. Zeolite Conf., 12th*, Vol. 3; Materials Research Society: 1999.

[57] Michael, J.; Kirchner, M. **1994,** *74,* 523–528.

[58] McCusker, L. B.; Baerlocher, C. *Microporous Mesoporous Mater.* **1996,** *6,* 51–54.

[59] Bennett, J.; Marcus, B. The Crystal Structures of Several Metal Aluminophosphate Molecular Sieves. In *Stud. Surf. Sci. Catal.*; Elsevier: 1988.

[60] McGuire, N. K.; Bateman, C. A.; Scott Blackwell, C.; Wilson, S. T.; Kirchner, R. M. *Zeolites* **1995,** *15,* 460–469.

[61] Broach, R. W.; Greenlay, N.; Jakubczak, P.; Knight, L. M.; Miller, S. R.; Mowat, J. P.; Stanczyk, J.; Lewis, G. J. *Microporous Mesoporous Mater.* **2014,** *189,* 49–63.

[62] Wilson, S. T.; McGuire, N. K.; Blackwell, C. S.; Bateman, C. A.; Kirchner, R. M. Synthesis, characterization, and structure of SAPO-56, a new member of the abc double-six ring family of materials with stacking sequence AABBCCBB. In *Zeolite Sci. 1994 Recent Prog. Discuss.*; 1995.

[63] Li, H.-x.; Davis, M. E.; Higginsb, J. B.; Dessaub, R. M. *J. Chem. Soc., Chem. Commun.* **1993,** *184,* 403–405.

[64] Pechar, F. *Zeolites* **1988,** *8,* 247–249.

[65] Vi, H. H.; Pluth, J.; Smith, J. V. **1986,** *42992,* 1118–1120.

[66] Keller, E. B.; Meier, W. M.; Kirchner, R. M. *Solid State Ionics* **1990,** *43,* 93–102.

[67] CAULLET, P.; GUTH, J. L.; HAZM, J.; LAMBLIN, J. M.; GIES, H. *ChemInform* **2010,** *22,* no–no.

[68] Michael Bennett, J.; Kirchner, R. M. *Zeolites* **1991,** *11,* 502–506.

[69] Li, H.; Yaghi, O. M. *J. Am. Chem. Soc.* **1998,** *120,* 10569–10570.

[70] Ojo, A. F.; McCusker, L. B. *Zeolites* **1991,** *11,* 460–465.

[71] Baur, W. H.; Joswig, W.; Kassner, D.; Kornatowski, J.; Finger, G. *Acta Crystallogr. Sect. B* **1994,** *50,* 290–294.

[72] Smith, J. V.; Pluth, J. J.; Andries, K. J. *Zeolites* **1993,** *13,* 166–169.

[73] Smeets, S.; McCusker, L. B.; Baerlocher, C.; Elomari, S.; Xie, D.; Zones, S. I. *J. Am. Chem. Soc.* **2016,** *138,* 7099–7106.

[74] Rudolf, P. R.; Saldarriaga-Molina, C.; Clearfield, A. *J. Phys. Chem.* **1986,** *90,* 6122–6125.

[75] Richardson, J. W.; Smith, J. V.; Pluth, J. J. *J. Phys. Chem.* **1990,** *94,* 3365–3367.

[76] Yuhas, B. D.; Mowat, J. P.; Miller, M. A.; Sinkler, W. *Chem. Mater.* **2018,** *30,* 582–586.

[77] Richardson, J. W.; Pluth, J. J.; Smith, J. V. *Naturwissenschaften* **1989,** *76,* 467–469.

[78] Dollase, W. A.; Ross, C. R. *Am. Mineral.* **1993,** *78,* 627–632.

[79] Conradsson, T.; Dadachov, M. S.; Zou, X. D. *Microporous Mesoporous Mater.* **2000,** *41,* 183–191.

[80] Corma, A.; Navarro, M. T.; Rey, F.; Rius, J.; Valencia, S. *Angew. Chemie - Int. Ed.* **2001,** *40,* 2277–2280.

[81] Ståhl, K.; Kvick, Å.; Ghose, S. *Zeolites* **1989,** *9,* 303–311.

[82] Bu, X.; Feng, P.; Gier, T. E.; Zhao, D.; Stucky, G. D. *J. Am. Chem. Soc.* **1998,** *120,* 13389–13397.

[83] Pluth, J. J.; Smith, J. V. *Am. Mineral.* **1990,** *75,* 501–507.

[84] Littlefield, B. T.; Weller, M. T. *Nat. Commun.* **2012,** *3*,.

[85] Harvey, G.; Baerlocher, C.; Wroblewski, T. *Zeitschrift für Krist. - Cryst. Mater.* **1992,** *201,* 113.

[86] Schlenker, J. L.; Pluth, J. J.; Smith, J. V. *Acta Crystallogr. Sect. B Struct. Crystallogr. Cryst. Chem.* **1977,** *33,* 2907–2910.

[87] Smolin, Y. I.; Shepelev, Y. F.; Butikova, I. K.; Kobyakov, I. B. *Kristallografiya* **1981,** *26,* 63–66.

[88] Peacor, D. R.; Rouse, R. C.; Ahn, J.-H. *Am. Mineral.* **1987,** *72,* 816–820.

[89] Araki, T. *Zeitschrift für Krist. - Cryst. Mater.* **1980,** *152,* 207.

[90] Knight, L. M.; Miller, M. A.; Koster, S. C.; Gatter, M. G.; Benin, A. I.; Willis, R. R.; Lewis, G. J.; Broach, R. W. UZM-13, UZM-17, UZM-19 and UZM-25: synthesis and structure of new layered precursors and a zeolite discovered via combinatorial chemistry techniques. In *From Zeolites to Porous MOF Mater. - 40th Anniv. Int. Zeolite Conf.*, Vol. 170; Xu, R.; Gao, Z.; Chen, J.; Yan, W., Eds.; Elsevier: 2007.

[91] Wagner, P.; Yoshikawa, M.; Lovallo, M.; Tsuji, K.; Taspatsis, M.; Davis, M. E. *Chem. Commun.* **1997,** 2179–2180.

[92] Chippindale, A. M.; Cowley, A. R. *Zeolites* **1997,** *18,* 176–181.

[93] Cowley, A. R.; Chippindale, A. M. *Microporous Mesoporous Mater.* **1999,** *28,* 163–172.

[94] Calligaris, M.; Nardin, G.; Randaccio, L. *Zeolites* **1983,** *3,* 205–208.

[95] Pluth, J. J.; Smith, J. V. *J. Phys. Chem.* **1989,** *93,* 6516–6520.

[96] Lobo, R. F.; Davis, M. E. *J. Am. Chem. Soc.* **1995,** *117,* 3766–3779.

[97] Schmidt, J. E.; Xie, D.; Rea, T.; Davis, M. E. *Chem. Sci.* **2015,** *6,* 1728–1734.

[98] Harrison, W. T.; Gier, T. E.; Stucky, G. D.; Broach, R. W.; Bedard, R. A. *Chem. Mater.* **1996,** *8,* 145–151.

[99] Vezzalini, G. *Zeitschrift für Krist. - Cryst. Mater.* **1984,** *166,* 63.

[100] Gies, H. *Zeitschrift für Krist. - Cryst. Mater.* **1986,** *175,* 93.

[101] Wright, P. A.; Jones, R. H.; Natarajan, S.; Bell, R. G.; Chen, J.; Hursthouse, M. B.; Thomas, J. M. *J. Chem. Soc. Chem. Commun.* **1993,** 633–635.

[102] Ove Kongshaug, K.; Fjellvag, H.; Petter Lillerud, K. *Chem. Mater.* **2000,** *12,* 1095–1099.

[103] Gerke, H.; Gies, H. *Zeitschrift für Krist. - Cryst. Mater.* **1984,** *166,* 11.

[104] Wessels, T.; Baerlocher, C.; McCusker, L. B.; Creyghton, E. J. *J. Am. Chem. Soc.* **1999,** *121,* 6242–6247.

[105] Rudinger, B.; Tillmanns, E.; Hentschel, G. *Mineral. Petrol.* **1993,** *48,* 147–152.

[106] Meier, W.; Groner, M. *J. Solid State Chem.* **1981,** *37,* 204–218.

[107] Kvick, Å.; Smith, J. V. *J. Chem. Phys.* **1983,** *79,* 2356–2362.

[108] Baerlocher, C.; Barker, R. M. *Zeitschrift für Krist. - Cryst. Mater.* **1974,** *140,* 10.

[109] Smeets, S.; Xie, D.; McCusker, L. B.; Baerlocher, C.; Zones, S. I.; Thompson, J. A.; Lacheen, H. S.; Huang, H. M. *Chem. Mater.* **2014,** *26,* 3909–3913.

[110] Baerlocher, C.; McCusker, L. B.; Chiappetta, R. *Microporous Mater.* **1994,** *2,* 269–280.

[111] Warrender, S. J.; Wright, P. A.; Zhou, W.; Lightfoot, P.; Camblor, M. A.; Shin, C. H.; Dong, J. K.; Suk, B. H. *Chem. Mater.* **2005,** *17,* 1272–1274.

[112] Perrotta, A. J. *Mineral. Mag. J. Mineral. Soc.* **1967,** *36,* 480–490.

[113] Gard, J. A.; Tait, J. M. No Title. In *Proc. 3rd Int. Conf. Mol. Sieves*; 1973.

[114] Pluth, J. J.; Smith, J. V.; Bennett, J. M. *Acta Crystallogr. Sect. C Cryst. Struct. Commun.* **1986,** *42,* 283–286.

[115] Millini, R.; Perego, G.; Carluccio, L.; Bellussi, G.; Cox, D. E.; Campbell, B. J.; Cheetham, A. K. The Synthesis of Zeolite ERS-7 and Its Structure Determination Using Simulated Annealing and Synchrotron X-ray Powder Diffraction. In *Int. zeolite Conf.*; 1999.

[116] Bae, J.; Cho, J.; Lee, J. H.; Seo, S. M.; Hong, S. B. *Angew. Chemie - Int. Ed.* **2016,** *55,* 7369–7373.

[117] Strohmaier, K. G.; Vaughan, D. E. W. *J. Am. Chem. Soc.* **2003,** *125,* 16035–16039.

[118] Briscoe, N.; Johnson, D.; Shannon, M.; Kokotailo, G.; McCusker, L. *Zeolites* **1988,** *8,* 74–76.

[119] Arranz, M.; Pérez-Pariente, J.; Wright, P. A.; Slawin, A. M.; Blasco, T.; Gómez-Hortigüela, L.; Cora, F. *Chem. Mater.* **2005,** *17,* 4374–4385.

[120] Guo, P.; Strohmaier, K.; Vroman, H.; Afeworki, M.; Ravikovitch, P. I.; Paur, C. S.; Sun, J.; Burton, A.; Zou, X. *Inorg. Chem. Front.* **2016,** *3,* 1444–1448.

[121] Afeworki, M.; Dorset, D. L.; Kennedy, G. J.; Strohmaier, K. G. *Chem. Mater.* **2006,** *18,* 1697–1704.

[122] Cá mara, F.; Bellatreccia, F.; Ventura, G. D.; Mottana, A. *Eur. J. Mineral.* **2006,** *17,* 839–846.

[123] Baur, W. H. *Am. Mineral.* **1964,** *49,* 697–704.

[124] Olson, D. H. *Zeolites* **1995,** *15,* 439–443.

[125] Parise, J. B.; Corbin, D. R.; Abrams, L.; Cox, D. E. *Acta Crystallogr. Sect. C Cryst. Struct. Commun.* **1984,** *40,* 1493–1497.

[126] Hriljac, J. A.; Eddy, M. M.; Cheetham, A. K.; Donohue, J. A.; Ray, G. J. *J. Solid State Chem.* **1993,** *106,* 66–72.

[127] Feuerstein, M.; Lobo, R. F. *Chem. Mater.* **1998,** *10,* 2197–2204.

[128] Morris, R. E.; Weigel, S. J.; Henson, N. J.; Bull, L. M.; Janicke, M. T.; Chmelka, B. F.; Cheetham, A. K. *J. Am. Chem. Soc.* **1994,** *116,* 11849–11855.

[129] Ballirano, P.; Bonaccorsi, E.; Maras, A.; Merlino, S. *Can. Mineral.* **2000,** *38,* 657–668.

[130] FISCHER, K. F.; SCHRAMM, V. Crystal Structure of Gismondite, a Detailed Refinement. In *Adv. Chem.*; 1974.

[131] Alberti, A.; Vezzalini, G. *Acta Crystallogr. Sect. B Struct. Crystallogr. Cryst. Chem.* **1979,** *35,* 2866–2869.

[132] McCusker, L. B.; Baerlocher, C.; Nawaz, R. *Zeitschrift für Krist. - Cryst. Mater.* **1985,** *171,* 281.

[133] Pluth, J. J.; Smith, J. V.; Bennett, J. M. *J. Am. Chem. Soc.* **1989,** *111,* 1692–1698.

[134] Baerlocher, C.; Meier, W. M. *Zeitschrift für Krist.* **1972,** *135,* 339–354.

[135] Bonaccorsi, E. *Microporous Mesoporous Mater.* **2004,** *73,* 129–136.

[136] Fischer, K. *Neues Jahrb. für Mineral. Monatshefte* **1966,** *1,* 13.

[137] Plévert, J.; Okubo, T.; Kubota, Y.; Honda, T.; Sugi, Y. *Chem. Commun.* **2000,** 2363–2364.

[138] Rouse, R. C.; Peacor, D. R. *Am. Mineral.* **1986,** *71,* 1494–1501.

[139] Merkle, A. B.; Slaughter, M. *Am. Mineral.* **1967,** *52,* 273–276.

[140] Koyama, K.; Takéuchi, Y. *Zeitschrift für Krist. - Cryst. Mater.* **1977,** *145,* 216.

[141] Martinez-Franco, R.; Moliner, M.; Yun, Y.; Sun, J.; Wan, W.; Zou, X.; Corma, A. *Proc. Natl. Acad. Sci.* **2013,** *110,* 3749–3754.

[142] Barrett, P. A.; Camblor, M. A.; Corma, A.; Jones, R. H.; Villaescusa, L. A. *Chem. Mater.* **1997,** *9,* 1713–1715.

[143] Simancas, R.; Jordá, J. L.; Rey, F.; Corma, A.; Cantín, A.; Peral, I.; Popescu, C. *J. Am. Chem. Soc.* **2014,** *136,* 3342–3345.

[144] Smeets, S.; McCusker, L. B.; Baerlocher, C.; Xie, D.; Chen, C.-Y.; Zones, S. I. *J. Am. Chem. Soc.* **2015,** *137,* 2015–2020.

[145] Jordá, J. L.; Rey, F.; Sastre, G.; Valencia, S.; Palomino, M.; Corma, A.; Segura, A.; Errandonea, D.; Lacomba, R.; Manjón, F. J.; Gomis, Ó.; Kleppe, A. K.; Jephcoat, A. P.; Amboage, M.; Rodríguez-Velamazán, J. A. *Angew. Chemie Int. Ed.* **2013,** *52,* 10458–10462.

[146] Cantín, A.; Corma, A.; Leiva, S.; Rey, F.; Rius, J.; Valencia, S. *J. Am. Chem. Soc.* **2005,** *127,* 11560–11561.

[147] Baerlocher, C.; Gramm, F.; Massuger, L.; McCusker, L. B.; He, Z.; Hovmoller, S.; Zou, X. *Science (80-. ).* **2007,** *315,* 1113–1116.

[148] Hernández-Rodríguez, M.; Jordá, J. L.; Rey, F.; Corma, A. *J. Am. Chem. Soc.* **2012,** *134,* 13232–13235.

[149] Jiang, J.; Jorda, J. L.; Diaz-Cabanas, M. J.; Yu, J.; Corma, A. *Angew. Chemie Int. Ed.* **2010,** *49,* 4986–4988.

[150] Villaescusa, L. A.; Barrett, P. A.; Camblor, M. A. *Angew. Chemie Int. Ed.* **1999,** *38,* 1997–2000.

[151] Camblor, M. A.; Corma, A.; Lightfoot, P.; Villaescusa, L. A.; Wright, P. A. *Angew. Chemie Int. Ed. English* **1997,** *36,* 2659–2661.

[152] Moliner, M.; Willhammar, T.; Wan, W.; González, J.; Rey, F.; Jorda, J. L.; Zou, X.; Corma, A. *J. Am. Chem. Soc.* **2012,** *134,* 6473–6478.

[153] Corma, A.; Puche, M.; Rey, F.; Sankar, G.; Teat, S. J. *Angew. Chemie Int. Ed.* **2003,** *42,* 1156–1159.

[154] Corma, A.; Diaz-Cabanas, M. J.; Jorda, J. L.; Rey, F.; Sastre, G.; Strohmaier, K. G. *J. Am. Chem. Soc.* **2008,** *130,* 16482–16483.

[155] Liu, L.; Yu, Z.-B.; Chen, H.; Deng, Y.; Lee, B.-L.; Sun, J. *Cryst. Growth Des.* **2013,** *13,* 4168–4171.

[156] Yang, X.; Camblor, M. A.; Lee, Y.; Liu, H.; Olson, D. H. *J. Am. Chem. Soc.* **2004,** *126,* 10403–10409.

[157] Castañeda, R.; Corma, A.; Fornés, V.; Rey, F.; Rius, J. *J. Am. Chem. Soc.* **2003,** *125,* 7820–7821.

[158] Dorset, D. L.; Strohmaier, K. G.; Kliewer, C. E.; Corma, A.; Dilaz-Cabanlas, M. J.; Rey, F.; Gilmore, C. J. *Chem. Mater.* **2008,** *20,* 5325–5331.

[159] Dorset, D. L.; Kennedy, G. J.; Strohmaier, K. G.; Diaz-Cabañas, M. J.; Rey, F.; Corma, A. *J. Am. Chem. Soc.* **2006,** *128,* 8862–8867.

[160] Corma, A.; Rey, F.; Valencia, S.; Jordá, J. L.; Rius, J. *Nat. Mater.* **2003,** *2,* 493–497.

[161] Rheinhardt, A.; Hellner, E.; Ahsbahs, H. *Fortsch. Miner.* **1982,** *60,* 175–176.

[162] Wang, Y.; Li, Y.; Yan, Y.; Xu, J.; Guan, B.; Wang, Q.; Li, J.; Yu, J. *Chem. Commun.* **2013,** *49,* 9006.

[163] Armstrong, J. A.; Weller, M. T. *J. Am. Chem. Soc.* **2010,** *132,* 15679–15686.

[164] Song, X.; Li, Y.; Gan, L.; Wang, Z.; Yu, J.; Xu, R. *Angew. Chemie Int. Ed.* **2009,** *48,* 314–317.

[165] Liu, Z.; Song, X.; Li, J.; Li, Y.; Yu, J.; Xu, R. *Inorg. Chem.* **2012,** *51,* 1969–1974.

[166] Xu, Y.; Li, Y.; Han, Y.; Song, X.; Yu, J. *Angew. Chemie Int. Ed.* **2013,** *52,* 5501–5503.

[167] Han, Y.; Li, Y.; Yu, J.; Xu, R. *Angew. Chemie Int. Ed.* **2011,** *50,* 3003–3005.

[168] Shao, L.; Li, Y.; Yu, J.; Xu, R. *Inorg. Chem.* **2012,** *51,* 225–229.

[169] Parise, J.; Shannon, R.; Prince, E.; Cox, D. *Zeitschrift für Krist. - Cryst. Mater.* **1983,** *165,* 175.

[170] Artioli, G.; Stahl, K. *Zeolites* **1993,** *13,* 249–255.

[171] Artioli, G.; Smith, J. V.; Kvick, Å. *Zeolites* **1989,** *9,* 377–391.

[172] Merlino, S.; Galli, E.; Alberti, A. *TMPM Tschermaks Mineral. und Petrogr. Mitteilungen* **1975,** *22,* 117–129.

[173] McCusker, L. *Mater. Sci. Forum* **1993,** *133-136,* 423–436.

[174] Merlino, S.; Orlandi, P. *Am. Mineral.* **1977,** *62,* 321–326.

[175] Schicker, P. *No Title,* Ph.D. thesis, ETH, Zurich, 1988.

[176] Merlino, S. *Eur. J. Mineral.* **1990,** *2,* 809–817.

[177] Gramlich, V.; MEIER, W. M. *Zeitschrift für Krist. - Cryst. Mater.* **1971,** *133,* 134.

[178] Pluth, J. J.; Smith, J. V. *J. Am. Chem. Soc.* **1980,** *102,* 4704–4708.

[179] McCusker, L. B.; Baerlocher, C.; Wilson, S. T.; Broach, R. W. *J. Phys. Chem. C* **2009,** *113,* 9838–9844.

[180] Broach, R. W.; Kirchner, R. M. *Microporous Mesoporous Mater.* **2011,** *143,* 398–400.

[181] Artioli, G.; Kvick, A. *Eur. J. Mineral.* **1990,** *2,* 749–759.

[182] Barrer, R. M.; Villiger, H. *Zeitschrift für Krist.* **1969,** *128,* 352–370.

[183] Shepelev, Y. F.; Smolin, Y. I.; Butikova, I. K.; Tarasov, V. I. *Dokl. Akad. Nauk SSSR* **1983,** *272,* 1133–1137.

[184] Bonaccorsi, E.; Orlandi, P. *Eur. J. Mineral.* **2004,** *15,* 1019–1027.

[185] Galli, E. *Rend. Soc. Ital. Miner. Pet.* **1974,** *31,* 599–612.

[186] Lawton, S. L.; Rohrbaugh, W. J. *Science (80-. ).* **1990,** *247,* 1319–1322.

[187] Fyfe, C. A.; Gies, H.; Kokotailo, G. T.; Pasztor, C.; Strobl, H.; Cox, D. E. *J. Am. Chem. Soc.* **1989,** *111,* 2470–2474.

[188] Terasaki, O.; Ohsuna, T.; Sakuma, H.; Watanabe, D.; Nakagawa, Y.; Medrud, R. C. *Chem. Mater.* **1996,** *8,* 463–468.

[189] Gies, H. *Zeitschrift für Krist. - Cryst. Mater.* **1983,** *164,* 247.

[190] Galli, E.; Gottardi, G.; Pongiluppi, D. *N. Jb. Miner. Mh.* **1979,** 1–9.

[191] Solov'eva, L. P.; Borisov, S. V.; Bakakin, V. V. *Sov. Phys. Crystallogr.* **1972,** *16,* 1035–1038.

[192] van Koningsveld, H.; van Bekkum, H.; Jansen, J. C. *Acta Crystallogr. Sect. B Struct. Sci.* **1987,** *43,* 127–132.

[193] VANKONINGSVELD, H.; JANSEN, J.; VANBEKKUM, H. *Zeolites* **1990,** *10,* 235–242.

[194] SCHLENKER, J.; HIGGINS, J.; VALYOCSIK, E. *Zeolites* **1990,** *10,* 293–296.

[195] Rouse, R. C.; Dunn, P. J.; Grice, J. D.; Schlenker, J. L.; Higgins, J. B. *Am. Mineral.* **1990,** *75,* 1415–1420.

[196] Rouse, R. C.; Peacor, D. R. *Am. Mineral.* **1994,** *79,* 175–184.

[197] Dorset, D. L. *Zeitschrift für Krist. - Cryst. Mater.* **2006,** *221,* 260–265.

[198] Willhammar, T.; Su, J.; Yun, Y.; Zou, X.; Afeworki, M.; Weston, S. C.; Vroman, H. B.; Lonergan, W. W.; Strohmaier, K. G. *Inorg. Chem.* **2017,** *56,* 8856–8864.

[199] Dorset, D. L.; Weston, S. C.; Dhingra, S. S. *J. Phys. Chem. B* **2006,** *110,* 2045–2050.

[200] Shantz, D. F.; Burton, A.; Lobo, R. F. *Microporous Mesoporous Mater.* **1999,** *31,* 61–73.

[201] Barrett, P. A.; Díaz-Cabañas, M.-J.; Camblor, M. A. *Chem. Mater.* **1999,** *11,* 2919–2927.

[202] Gies, I. *Zeitschrift für Krist. - Cryst. Mater.* **1984,** *167,* 73–82.

[203] Marler, B.; Deroche, C.; Gies, H.; Fyfe, C. A.; Grondey, H.; Kokotailo, G. T.; Feng, Y.; Ernst, S.; Weitkamp, J.; Cox, D. E. *J. Appl. Crystallogr.* **1993,** *26,* 636–644.

[204] Fyfe, C. A.; Gies, H.; Kokotailo, G. T.; Marler, B.; Cox, D. E. *J. Phys. Chem.* **1990,** *94,* 3718–3721.

[205] Xie, D.; McCusker, L. B.; Baerlocher, C.; Gibson, L.; Burton, A. W.; Hwang, S.-J. *J. Phys. Chem. C* **2009,** *113,* 9845–9850.

[206] Guo, P.; Shin, J.; Greenaway, A. G.; Min, J. G.; Su, J.; Choi, H. J.; Liu, L.; Cox, P. A.; Hong, S. B.; Wright, P. A.; Zou, X. *Nature* **2015,** *524,* 74–78.

[207] Camblor, M. A.; Corma, A.; Díaz-Cabañas, M.-J.; Baerlocher, C. *J. Phys. Chem. B* **1998,** *102,* 44–51.

[208] Petersen, O. V.; Giester, G.; Brandstatter, F.; Niedermayr, G. *Can. Mineral.* **2002,** *40,* 173–181.

[209] Meier, W. M. *Zeitschrift für Krist.* **1960,** *113,* 430–444.

[210] Artioli, G.; Smith, J. V.; Pluth, J. J. *Acta Crystallogr. Sect. C Cryst. Struct. Commun.* **1986,** *42,* 937–942.

[211] Smith, J. V.; Pluth, J. J.; Artioli, G.; Ross, F. K. Neutron and x-ray refinements of scolecite.. In *Proc. Int. Zeolite Conf., 6th*; Butterworth: 1984.

[212] Shannon, M. D.; Casci, J. L.; Cox, P. A.; Andrews, S. J. *Nature* **1991,** *353,* 417–420.

[213] Marler, B.; Dehnbostel, N.; Eulert, H. H.; Gies, H.; Liebau, F. *J. Incl. Phenom.* **1986,** *4,* 339–349.

[214] Correll, S.; Oeckler, O.; Stock, N.; Schnick, W. *Angew. Chemie Int. Ed.* **2003,** *42,* 3549–3552.

[215] Sedlmaier, S. J.; Doblinger, M.; Oeckler, O.; Weber, J.; Schmedt auf der Günne, J.; Schnick, W. *J. Am. Chem. Soc.* **2011,** *133,* 12069–12078.

[216] Zanardi, S.; Alberti, A.; Cruciani, G.; Corma, A.; Fornés, V.; Brunelli, M. *Angew. Chemie Int. Ed.* **2004,** *43,* 4933–4937.

[217] Cheetham, A.; Fjellvg, H.; Gier, T.; Kongshaug, K.; Lillerud, K.; Stucky, G. 05-O-05-Very open microporous materials: from concept to reality. In *Stud. Surf. Sci. Catal.*, Vol. 135; Elsevier: 2001.

[218] Alberti, A. *Zeolites* **1996,** *17,* 457–461.

[219] Verheyen, E. *et al. Nat. Mater.* **2012,** *11,* 1059–1064.

[220] Akporiaye, D. E.; Fjellvag, H.; Halvorsen, E. N.; Haug, T.; Karlsson, A.; Lillerud, K. P. *Chem. Commun.* **1996,** *1,* 1553–1554.

[221] Kongshaug, K. O.; Fjellvåg, H.; Lillerud, K. P. *J. Mater. Chem.* **2001,** *11,* 1242–1247.

[222] Gordon, E. K.; Samson, S.; Kamb, W. B. *Science (80-. ).* **1966,** *154,* 1004–1007.

[223] Roth, W. J.; Nachtigall, P.; Morris, R. E.; Wheatley, P. S.; Seymour, V. R.; Ashbrook, S. E.; Chlubná, P.; Grajciar, L.; Položij, M.; Zukal, A.; Shvets, O.; Čejka, J. *Nat. Chem.* **2013,** *5,* 628–633.

[224] Rinaldi, R.; Pluth, J. J.; Smith, J. V. *Acta Crystallogr. Sect. B Struct. Crystallogr. Cryst. Chem.* **1974,** *30,* 2426–2433.

[225] Jordá, J. L.; McCusker, L. B.; Baerlocher, C.; Morais, C. M.; Rocha, J.; Fernandez, C.; Borges, C.; Lourenço, J. P.; Ribeiro, M. F.; Gabelica, Z. *Microporous Mesoporous Mater.* **2003,** *65,* 43–57.

[226] Seo, S.; Yang, T.; Shin, J.; Jo, D.; Zou, X.; Hong, S. B. *Angew. Chemie Int. Ed.* **2018,** *57,* 3727–3732.

[227] Hua, W.; Chen, H.; Yu, Z.-B.; Zou, X.; Lin, J.; Sun, J. *Angew. Chemie Int. Ed.* **2014,** *53,* 5868–5871.

[228] Lee, J. K.; Turrina, A.; Zhu, L.; Seo, S.; Zhang, D.; Cox, P. A.; Wright, P. A.; Qiu, S.; Hong, S. B. *Angew. Chemie Int. Ed.* **2014,** *53,* 7480–7483.

[229] Su, J.; Wang, Y.; Wang, Z.; Lin, J. *J. Am. Chem. Soc.* **2009,** *131,* 6080–6081.

[230] Lee, H.; Shin, J.; Choi, W.; Choi, H. J.; Yang, T.; Zou, X.; Hong, S. B. *Chem. Mater.* **2018,** *30,* 6619–6623.

[231] Jo, D.; Park, G. T.; Shin, J.; Hong, S. B. *Angew. Chemie Int. Ed.* **2018,** *57,* 2199–2203.

[232] Rouse, R. C.; Peacor, D. R.; Merlino, S. *Am. Mineral.* **1989,** *74,* 1195–1202.

[233] McCusker, L. B.; Baerlocher, C. The effect of dehydration upon the crystal structure of zeolite rho.. In *Proc. Int. Zeolite Conf., 6th*; Butterworth: 1984.

[234] Parise, K. B.; Corbin, D. R.; Gier, T. E.; Harlow, R. L.; Abrams, L.; Von Dreele, R. B. *Zeolites* **1992,** *12,* 360–368.

[235] Wang, Y. X.; Gies, H.; Marler, B.; Müller, U. *Chem. Mater.* **2005,** *17,* 43–49.

[236] Röhrig, C.; Gies, H. *Angew. Chemie Int. Ed. English* **1995,** *34,* 63–65.

[237] Marler, B.; Grünewald-Lüke, A.; Gies, H. *Zeolites* **1995,** *15,* 388–399.

[238] Vortmann, S.; Marler, B.; Gies, H.; Daniels, P. *Microporous Mater.* **1995,** *4,* 111–121.

[239] Gies, H.; Rius, J. *Zeitschrift für Krist. - Cryst. Mater.* **1995,** *210,* 475–480.

[240] Marler, B.; Ströter, N.; Gies, H. *Microporous Mesoporous Mater.* **2005,** *83,* 201–211.

[241] Zheng, N. *Science (80-. ).* **2002,** *298,* 2366–2369.

[242] Han, Z.; Picone, A. L.; Slawin, A. M.; Seymour, V. R.; Ashbrook, S. E.; Zhou, W.; Thompson, S. P.; Parker, J. E.; Wright, P. A. *Chem. Mater.* **2010,** *22,* 338–346.

[243] Noble, G. W.; Wright, P. A.; Lightfoot, P.; Morris, R. E.; Hudson, K. J.; Kvick, Å.; Graafsma, H. *Angew. Chemie Int. Ed. English* **1997,** *36,* 81–83.

[244] Patinec, V.; Wright, P. A.; Lightfoot, P.; Aitken, R. A.; Cox, P. A. *J. Chem. Soc. Dalt. Trans.* **1999,** 3909–3911.

[245] Noble, G. W.; Wright, P. A.; Kvick, Å. *J. Chem. Soc. - Dalt. Trans.* **1997,** *23,* 4485–4490.

[246] Wright, P. A.; Maple, M. J.; Slawin, A. M. Z.; Patinec, V.; Aitken, R. A.; Welsh, S.; Cox, P. A. *J. Chem. Soc. Dalt. Trans.* **2000,** 1243–1248.

[247] Bu, X. *Science (80-. ).* **1997,** *278,* 2080–2085.

[248] Shi, L.; Christensen, K. E.; Jansson, K.; Sun, J.; Zou, X. *Chem. Mater.* **2007,** *19,* 5973–5979.

[249] Xie, D.; McCusker, L. B.; Baerlocher, C. *J. Am. Chem. Soc.* **2011,** *133,* 20604–20610.

[250] Wagner, P.; Terasaki, O.; Ritsch, S.; Nery, J. G.; Zones, S. I.; Davis, M. E.; Hiraga, K. *J. Phys. Chem. B* **1999,** *103,* 8245–8250.

[251] Wagner, P.; Zones, S. I.; Davis, M. E.; Medrud, R. C. *Angew. Chemie Int. Ed.* **1999,** *38,* 1269–1272.

[252] Burton, A.; Elomari, S.; Medrud, R. C.; Chan, I. Y.; Chen, C.-Y.; Bull, L. M.; Vittoratos, E. S. *J. Am. Chem. Soc.* **2003,** *125,* 1633–1642.

[253] Burton, A.; Elomari, S.; Chen, C.-Y.; Medrud, R. C.; Chan, I. Y.; Bull, L. M.; Kibby, C.; Harris, T. V.; Zones, S. I.; Vittoratos, E. S. *Chem. - A Eur. J.* **2003,** *9,* 5737–5748.

[254] Morris, R. E.; Burton, A.; Bull, L. M.; Zones, S. I. *Chem. Mater.* **2004,** *16,* 2844–2851.

[255] Elomari, S.; Burton, A.; Medrud, R. C.; Grosse-Kunstleve, R. *Microporous Mesoporous Mater.* **2009,** *118,* 325–333.

[256] Xie, D.; McCusker, L. B.; Baerlocher, C.; Zones, S. I.; Wan, W.; Zou, X. *J. Am. Chem. Soc.* **2013,** *135,* 10519–10524.

[257] McCusker, L. *J. Appl. Crystallogr.* **1988,** *21,* 305–310.

[258] Parnham, E. R.; Morris, R. E. *J. Am. Chem. Soc.* **2006,** *128,* 2204–2205.

[259] Felsche, J.; Luger, S.; Baerlocher, C. *Zeolites* **1986,** *6,* 367–372.

[260] SAHL, K.; CHATTERJEE, N. D. *Zeitschrift für Krist. - Cryst. Mater.* **1978,** *146,* 35–42.

[261] Hassan, I.; Grundy, H. D.; Ls, O.; Dand, A. C. **1991,** 385–390.

[262] Tang, L.; Shi, L.; Bonneau, C.; Sun, J.; Yue, H.; Ojuva, A.; Lee, B.-L.; Kritikos, M.; Bell, R. G.; Bacsik, Z.; Mink, J.; Zou, X. *Nat. Mater.* **2008,** *7,* 381–385.

[263] Luo, Y.; Smeets, S.; Peng, F.; Etman, A. S.; Wang, Z.; Sun, J.; Yang, W. *Chem. - A Eur. J.* **2017,** *23,* 16829–16834.

[264] Li, Y.; Zou, X. *Angew. Chemie Int. Ed.* **2005,** *44,* 2012–2015.

[265] Luo, Y.; Smeets, S.; Wang, Z.; Sun, J.; Yang, W. *Chem. - A Eur. J.* **2019,** *25,* 2184–2188.

[266] Elomari, S.; Burton, A. W.; Ong, K.; Pradhan, A. R.; Chan, I. Y. *Chem. Mater.* **2007,** *19,* 5485–5492.

[267] Burton, A.; Elomari, S. *Chem. Commun.* **2004,** *10,* 2618.

[268] Galli, E. *Acta Crystallogr. Sect. B Struct. Crystallogr. Cryst. Chem.* **1971,** *27,* 833–841.

[269] Galli, E.; Alberti, A. *Bull. la Société française Minéralogie Cristallogr.* **1975,** *98,* 11–18.

[270] Galli, E.; Alberti, A. *Bull. la Société française Minéralogie Cristallogr.* **1975,** *98,* 331–340.

[271] Camblor, M. A.; Díaz-Cabañas, M.-J.; Perez-Pariente, J.; Teat, S. J.; Clegg, W.; Shannon, I. J.; Lightfoot, P.; Wright, P. A.; Morris, R. E. *Angew. Chemie Int. Ed.* **1998,** *37,* 2122–2126.

[272] McCusker, L. B.; Baerlocher, C.; Burton, A. W.; Zones, S. I. *Solid State Sci.* **2011,** *13,* 800–805.

[273] Turrina, A.; Garcia, R.; Watts, A. E.; Greer, H. F.; Bradley, J.; Zhou, W.; Cox, P. A.; Shannon, M. D.; Mayoral, A.; Casci, J. L.; Wright, P. A. *Chem. Mater.* **2017,** *29,* 2180–2190.

[274] Strohmeier, K. G.; Afeworki, M.; Dorset, D. L. *Zeitschrift für Krist. - Cryst. Mater.* **2006,** *221,* 689–698.

[275] Galli, E.; Quartieri, S.; Vezzalini, G.; Alberti, A.; Franzini, M. *Am. Mineral.* **1997,** *82,* 423–429.

[276] Pluth, J. J.; Smith, J. V.; Kvick, Å. *Zeolites* **1985,** *5,* 74–80.

[277] Rozenberg, K. A.; Sapozhnikov, A. N.; Rastsvetaeva, R. K.; Bolotina, N. B.; Kashaev, A. A. *Crystallogr. Reports* **2004,** *49,* 635–642.

[278] Marler, B. *Zeolites* **1987,** *7,* 393–397.

[279] Effenberger, H.; Giester, G.; Krause, W.; Bernhardt, H. J. *Am. Mineral.* **1998,** *83,* 607–617.

[280] Gramm, F.; Baerlocher, C.; McCusker, L. B.; Warrender, S. J.; Wright, P. A.; Han, B.; Hong, S. B.; Liu, Z.; Ohsuna, T.; Terasaki, O. *Nature* **2006,** *444,* 79–81.

[281] Josien, L.; Simon, A.; Gramlich, V.; Patarin, J. *Chem. Mater.* **2001,** *13,* 1305–1311.

[282] Blackwell, C. S. *et al. Angew. Chemie Int. Ed.* **2003,** *42,* 1737–1740.

[283] Lorgouilloux, Y.; Dodin, M.; Paillaud, J.-L.; Caullet, P.; Michelin, L.; Josien, L.; Ersen, O.; Bats, N. *J. Solid State Chem.* **2009,** *182,* 622–629.

[284] Lorgouilloux, Y.; Dodin, M.; Mugnaioli, E.; Marichal, C.; Caullet, P.; Bats, N.; Kolb, U.; Paillaud, J.-L. *RSC Adv.* **2014,** *4,* 19440.

[285] Mathieu, Y.; Paillaud, J.-L.; Caullet, P.; Bats, N. *Microporous Mesoporous Mater.* **2004,** *75,* 13–22.

[286] Josien, L.; Simon-Masseron, A.; Gramlich, V.; Patarin, J.; Rouleau, L. *Chem. - A Eur. J.* **2003,** *9,* 856–861.

[287] Paillaud, J.-L. *Science (80-. ).* **2004,** *304,* 990–992.

[288] Dodin, M.; Paillaud, J.-L.; Lorgouilloux, Y.; Caullet, P.; Elkailm, E.; Bats, N. *J. Am. Chem. Soc.* **2010,** *132,* 10221–10223.

[289] Freyhardt, C. C.; Lobo, R. F.; Khodabandeh, S.; Lewis, J. E.; Tsapatsis, M.; Yoshikawa, M.; Camblor, M. A.; Pan, M.; Helmkamp, M. M.; Zones, S. I.; Davis, M. E. *J. Am. Chem. Soc.* **1996,** *118,* 7299–7310.

[290] McCusker, L.; Baerlocher, C.; Jahn, E.; Bülow, M. *Zeolites* **1991,** *11,* 308–313.

[291] Röhrig, C.; Gies, H.; Marler, B. *Zeolites* **1994,** *14,* 498–503.

[292] Walter, F. *Eur. J. Mineral.* **1992,** *4,* 1275–1283.

[293] Nakazawa, N.; Ikeda, T.; Hiyoshi, N.; Yoshida, Y.; Han, Q.; Inagaki, S.; Kubota, Y. *J. Am. Chem. Soc.* **2017,** *139,* 7989–7997.

[294] Kvick, Å.; Artioli, G.; Smith, J. V. *Zeitschrift für Krist. - Cryst. Mater.* **1986,** *174,* 265–282.

[295] Marler, B.; Patarin, J.; Sierra, L. *Microporous Mater.* **1995,** *5,* 151–159.

[296] Arnold, G. P.; Finch, E. D.; Rabideau, S. W.; Wenzel, R. G. *J. Chem. Phys.* **1968,** *49,* 4365–4369.

[297] Goto, A.; Hondoh, T.; Mae, S. *J. Chem. Phys.* **1990,** *93,* 1412–1417.

[298] Kamb, B. *Acta Crystallogr.* **1964,** *17,* 1437–1449.

[299] Iii, I. *Nature* **1936,** *137,* 327.

[300] Engelhardt, H.; Kamb, B. *J. Chem. Phys.* **1981,** *75,* 5887–5899.

[301] Kamb, B.; Prakash, A.; Knobler, C. *Acta Crystallogr.* **1967,** *22,* 706–715.

[302] Kuhs, W. F.; Finney, J. L.; Vettier, C.; Bliss, D. V. *J. Chem. Phys.* **1984,** *81,* 3612–3623.

[303] Kamb, B.; Davis, B. L. *Proc. Natl. Acad. Sci.* **1964,** *52,* 1433–1439.

[304] Besson, J. M.; Pruzan, P.; Klotz, S.; Hamel, G.; Silvi, B.; Nelmes, R. J.; Loveday, J. S.; Wilson, R. M.; Hull, S. *Phys. Rev. B* **1994,** *49,* 12540–12550.

[305] La Placa, S. J.; Hamilton, W. C.; Kamb, B.; Prakash, A. *J. Chem. Phys.* **1973,** *58,* 567–580.

[306] Fukazawa, H.; Hoshikawa, A.; Yamauchi, H.; Yamaguchi, Y.; Ishii, Y. *J. Cryst. Growth* **2005,** *282,* 251–259.

[307] Koza, M.; Schober, H.; Tölle, A.; Fujara, F.; Hansen, T. *Nature* **1999,** *397,* 660–661.

[308] Salzmann, C. G.; Radaelli, P. G.; Hallbrucker, A.; Mayer, E.; Finney, J. L. *Phys. Chem. Ice* **2007,** *311,* 521–528.

[309] Salzmann, C. G.; Radaelli, P. G.; Mayer, E.; Finney, J. L. *Phys. Rev. Lett.* **2009,** *103,* 1–4.

[310] Falenty, A.; Hansen, T. C.; Kuhs, W. F. *Nature* **2014,** *516,* 231–233.

[311] Del Rosso, L.; Grazzi, F.; Celli, M.; Colognesi, D.; Garcia-Sakai, V.; Ulivi, L. *J. Phys. Chem. C* **2016,** *120,* 26955–26959.

[312] Lapidus, S. H.; Halder, G. J.; Chupas, P. J.; Chapman, K. W. *J. Am. Chem. Soc.* **2013,** *135,* 7621–7628.

[313] Kirchner, M. T.; Boese, R.; Billups, W. E.; Norman, L. R. *J. Am. Chem. Soc.* **2004,** *126,* 9407–9412.

[314] Mootz, D.; josef Oellers, E.; Wiebcke, M. *J. Am. Chem. Soc.* **1987,** *109,* 1200–1202.

[315] McMullan, R. K.; Jeffrey, G. A. *J. Chem. Phys.* **1965,** *42,* 2725–2732.

[316] McMullan, R. K.; Kvick, *Acta Crystallogr. Sect. B* **1990,** *46,* 390–399.

[317] Troyanov, S. I. *Zhurnal Neorg. Khimii* **2000,** *45,* 1619–1624.

[318] Brehler, B. *Zeitschrift für Krist.* **1961,** *115,* 373–402.

[319] Evers, J.; Mayer, P.; Möckl, L.; Oehlinger, G.; Köppe, R.; Schnöckel, H. *Inorg. Chem.* **2015,** *54,* 1240–1253.

[320] Klüfers, P. *Zeitschrift für Krist. - Cryst. Mater.* **1984,** *167,*.

[321] "LiCo(CO)4 (LiCo[CO]4) Crystal Structure: Datasheet from "PAULING FILE Multinaries Edition â 2012" in SpringerMaterials (https://materials.springer.com/isp/crystallographic/docs/sd_1712939)".
